# An update of the reported effects of the COVID-19 pandemic on person with intellectual disability and their carers: a scoping review

**DOI:** 10.1080/07853890.2023.2196437

**Published:** 2023-04-18

**Authors:** Paul Michael Keenan, Owen Doody

**Affiliations:** a School of Nursing and Midwifery, Trinity College Dublin, Dublin, Ireland; b Department of Nursing and Midwifery, Health Research Institute, University of Limerick, Limerick, Ireland

**Keywords:** COVID-19, pandemic, intellectual disability, scoping review

## Abstract

**Background:**

The effects of the COVID-19 pandemic has been felt by all groups in society and people with intellectual disability are especially vulnerable due to underlying conditions/health problems, multi-morbidity, limitations in understanding, frailty and social circumstances. This places people with intellectual disability, their families and carers at increased risk of stress and in need of support.

**Objective:**

To update and chart the evidence of the effects of the COVID-19 pandemic on people with intellectual disability, their families and carers reported within the research in 2021.

**Methods:**

A scoping review of research published in 2021 across 7 databases.

**Results:**

84 studies met the inclusion criteria, and the findings highlight people with intellectual disability are at a greater risk to COVID-19 health outcomes due to underlying health concerns and access issues. The effects of COVID-19 can be seen from a personal, social and health perspective for people with intellectual disability, their carers and families. However, COVID-19 did have some unanticipated benefits such as: less demand on time, greater opportunity to engage with people of value and building resilience.

**Conclusions:**

COVID-19 presents many challenges but for people with intellectual disability compounding existing obstacles encountered in access issues, service provision and supports available. There is a need to identify and describe the experiences of people with intellectual disability, their families and carers in the medium-long term during COVID-19. Greater supports and evidence of effective interventions to promote health, deliver services and support individual with intellectual disability is needed as there is little evidence of clinical care for people with intellectual disability during COVID-19.

## Key Messages

- During pandemics the perspectives of people with intellectual disability, their carers and service providers are central to addressing systemic health care inequalities and poor-quality person-centred care.
- Greater collaboration is needed to learn from pandemics in terms of health and social care policy improvements.
- There remains a need for large scale studies that are representative of the broad spectrum of the intellectual disability population and examine Long-COVID in this group of people.

## Introduction

Since the virus underlying the COVID-19 disease, SARS-CoV-2 was first diagnosed in December 2019 in Wuhan, China, the world continues to experience a life-threatening viral pandemic whose future remains dangerously unpredictable [1]. As of 16th November 2022, there have been over 633 million confirmed cases of COVID-19, including 6,596,542 deaths reported to WHO and over 12.9 billion vaccine doses administered [2]. Over the past two and a half years national governments and public health bodies have advised the public to adopt responsive care [3]. Legal and health strategies have included guidelines on hand and respiratory hygiene, wearing face masks, social distancing, avoidance of gatherings and vaccination programmes [4]. National and regional screening, lockdown and remaining at home strategies have also been implemented to manage the disease’s transmission [5], with various success. In addition, the scientific community has moved to inform, support and monitor the international attempts to deal with the COVID-19 crisis [6].

There is a continued need for further research on Covid-19 and people with intellectual disability. The limited studies that exist are small scale and not representative of the broad spectrum of the intellectual disability population. Furthermore, many studies were mainly undertaken in the early stages of the pandemic therefore the medium-long term effects of COVID-19 remains poorly understood. However, the existing literature informs us that the COVID-19 pandemic has reinforced and expanded the healthcare inequalities historically experienced by people with intellectual disability and is significantly contributing to an increase in their existing poor health outcomes. COVID-19 has exacerbated and heightened the experience of such existing vulnerabilities for people with intellectual disability. Vulnerabilities include increased physical, mental and social health issues; increased mortality rate; higher risk of developing serious illness due to limitations in understanding of COVID-19 risk reduction strategies and restricted capacity to social distance within congregate care settings as they depend on others for support with their everyday needs [7].

Understanding the effects of COVID-19 on people with intellectual disability, their families and carers are central to ensuring person centred support to manage their needs during this prolonged pandemic and into the future. This is reinforced by the need for an ongoing process of evaluation and review of evolving evidence, for the continual reaction to the current ongoing pandemic and to inform policy decisions concerning possible future pandemics. This paper aims to update and build on the initial seminal review conducted by Doody and Keenan [7] to update and chart the evidence of the effects of the COVID-19 pandemic on people with intellectual disability, their families and carers. By reviewing the literature on the second year of the COVID-19 pandemic, the paper identifies what professions and organizations serving people with intellectual disability can learn from the first two years of COVID-19 to enhance preparedness, detection and response in the future. Within this paper, intellectual disability is characterized by impaired intellectual functioning and learned behaviour, which affects various everyday social and practical competencies [8].

## Methods

A scoping review methodological approach was selected as it permits the presentation of a broad synthesis and mapping of the available literature focused on the question under investigation, not limited by study quality or design [9]. Consequently, enabling the identification of the current body of knowledge and gaps in the literature [10]. Utilizing a scoping review facilitates a systematic and transparent synthesis of the evidence and a rigorous map of the findings to present the extent and gravity of the literature, identify gaps and make recommendations [11]. Arksey and O’Malley framework [9] was adopted integrating updates by Levac et al. [12] and Bradbury-Jones et al. [11]. Arksey and O’Malley framework [9] utilizing a five-step process: (i) identifying the research question, (ii) identifying relevant studies, (iii) study choice, (iv) plotting the data, and (v) arranging, summarizing and communicating the outcomes and is an interactive process where each step was returned to and advanced during the process. Results are conveyed as a narrative in conjunction with tables and diagram illustrations [10].

### Identification of the research question

To meet the aim of this review and ensure this update can be mapped to the original 2020 review the authors readdressed the original questions: (a) What effects are reported by people with intellectual disability and their carers? (b) What responses have been directed towards people with intellectual disability and their carers? and (c) What recommendations have been made regarding people with intellectual disability and their carers?

### Identification of relevant studies

Given that this paper is an update to an existing review the original search process was adopted and replicated from the original 2020 search which utilized broad terms for intellectual disability and COVID-19 (Table 1). This rigorous research approach is deemed fit for purpose. Searches were conducted utilizing and selecting the Title OR Abstract and Keyword options. Each search string was searched individually and subsequent combined. Inclusion and exclusion criteria were created in advance (Table 2) and utilized to uncover relevant papers across the seven electronic databases (CINAHL, Academic Search Complete, MEDLINE, PsycINFO, EMBASE, Scopus, Cochrane). Within the screening process for inclusion within this review, research papers were assessed firstly, against having a specifically identified study sample as having intellectual disability and secondly, against the broad definition identified earlier in the introduction of this paper.

**Table 1.** Search terms.

S1 = Epidemic OR Pandemic OR COVID OR COVID-19 OR Coronavirus
S2 = Intellectual disability* OR learning disability* OR developmental disability*
S3 = S1 + S2

* represents - Truncation used to broaden search to include various word endings and spellings.

**Table 2.** Inclusion/exclusion criteria.

Include	Exclude
Papers addressing information pertaining to coronavirus. Paper focus is on persons with intellectual disability and/or their carers. Papers published in 2021. English language. Primary peer reviewed research papers in journals.	Papers that do not addresses information pertaining to coronavirus. Papers where it is not possible to extract data focusing on persons with intellectual disability and/or their carers. Papers publisher prior to 2021 or after 2021 Non-English language paper. Non-primary research such as reports, working papers/protocols, conference abstracts, government and non-governmental organizations documents, opinion papers, letters to the editor, discussion papers, correspondence papers

### Study selection

**Figure 1.**
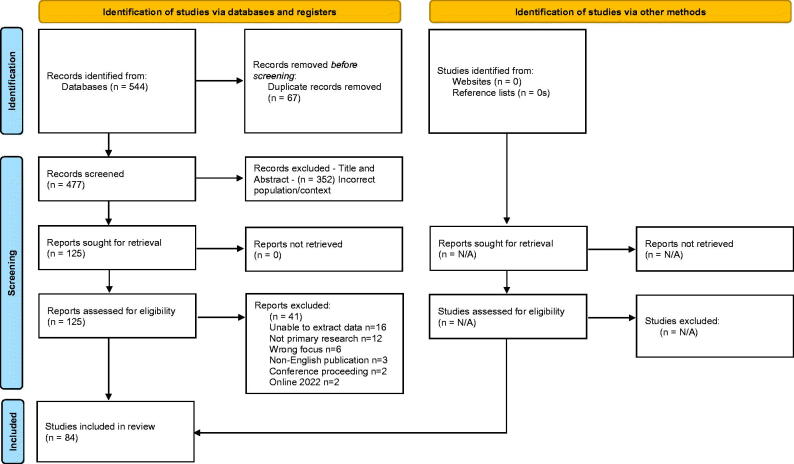
PRISMA flow diagram.

The search of the seven electronic databases yielded 544 papers which were transferred to Endnote and duplicates removed (*n* = 67) resulting in 477 papers remaining. Following this screening was conducted independently within Rayyan (Qatar Computing Research Institute) a web-tool for researchers working on reviews or synthesis projects to support the process of screening and selecting studies. Screening by the authors was guided by the inclusion criteria (Table 2). Initial screening of title and abstracts resulted in 352 papers being excluded and the remaining 125 papers were proceeded to full text review where they were assessed against the inclusion criteria. The authors then met to discuss remaining papers and form agreement, resulting in 42 papers being excluded and 84 meeting the criteria and therefore included in this review. A critical appraisal and risk of bias are not deemed essential and considered a choice in a scoping review [13] and in this review were not conducted as they were not deemed necessary to meet the aim and objectives of this review. The selection and reporting process followed Tricco et al. [14] Preferred Reporting Items for Scoping Reviews (PRISMA-Sc-R) and PRISMA flow diagram [15] (Figure 1). Eight papers were included as they were returned as 2021 publications, being available online between June and December 2021. However, journal assignment to hard copy has occurred post January 2022, thus when finalising this paper, a 2022 reference was utilized, but the papers were included as they were in the public arena and met the inclusion criteria when the review and screening process were conducted.

### Plotting the data

This scoping review aims to map the existing literature in terms of volume, nature, characteristics, and sources of evidence [16]. This involved plotting and charting the data and extracting synopses from each paper and details were included in a data extraction table (Table 3).

**Table 3.** Data extraction.

Author(s), Year, Title, Place	Aim	Methodology, population, methods	Summary of findings	Key messages	Limitations
Abuzaid, (2021) [17] Consequences of Coronavirus as a predictor of emotional security among mothers of children with Intellectual Disabilities. Saudi Arabia.	To explore the consequences of Coronavirus as a predictor of emotional security among mothers of children with intellectual disability (ID).	Quantitative – survey research. Sample −120 mothers. Data collection – online survey. Data analysis – descriptive and inferential statistics. Timeline – not identified.	Findings indicate that the greater the consequences of Coronavirus, the less emotionally secure the mother. is likely to feel during the COVID-19 lockdown in Saudi Arabia.	The lack of supports from services, family and the community for those who need it most negatively impacted the mothers.	Self-report measures used. Online surveys may be limited to those with access and connectivity and literacy issues may affect response rate.
Amor et al. (2021) [18] Perceptions of people with intellectual and developmental disabilities about COVID-19 in Spain: a cross-sectional study. Spain.	To explore the perceptions of Spanish people with ID on the effects of COVID-19 and the subsequent lockdown on their lives.	Quantitative – survey research. Sample − 582 people with ID. Data collection – online survey. Data analysis – descriptive and inferential statistics. Timeline – March/June 2020.	The pandemic and subsequent lockdown had a negative effect on emotional well-being. Access to information and support was reportedly good overall, being under the age of 21 years and studying were associated with perceptions reflecting poorer access to information and well-being support. Being supported by a third party to complete the survey was consistently related to perceptions of worse outcomes.	The pandemic and subsequent lockdown have had a very negative effect on participants	Self-report measures used. Online questionnaire may be limited to those with access and connectivity and literacy issues may affect response rate. Data collected very early in the pandemic and further data required to identify the long-term impact. While the number of participants was large, the sample was not stratified.
Araten-Bergman and Shpigelman (2021) [19] Staying connected during COVID-19: Family engagement with adults with developmental disabilities in supported accommodation. Australia.	To explore how family caregivers have interacted with and supported their relatives with ID residing in supported accommodation during the pandemic.	Quantitative –survey research. Sample − 108 family caregivers. Data collection - online survey. Data analysis – descriptive and inferential statistics. Timeline – April/May 2020.	Most family caregivers adopted remote communication technologies. These were not perceived as effective as compared to face-to-face contact. Families were able to provide emotional support and advocacy using digital technologies, however, were limited in their ability to provide social support.	Findings may help key stakeholders develop and implement novel strategies and policies to accommodate the changing circumstances and to ensure continuity of family engagement and informal support in the context of COVID-19.	Self-report measures used. Online questionnaire may be limited to those with access and connectivity and literacy issues may affect response rate. Data collected very early in the pandemic and further data required to identify the long-term impact.
Ashworth et al. (2021) [20] Online and face-to-face performance on two cognitive tasks in children with Williams Syndrome. United Kingdom.	To evaluate the appropriability and comparability of conducting online cognitive assessments (RCPM, BPVS) with children with Williams Syndrome.	Quantitative –survey research. Sample − 26 children with Williams Syndrome (14 online, 12 face-to-face). Data collection – online survey. Data analysis – descriptive and inferential statistics. Timeline – not identified.	Bayesian t-tests showed that children’s RCPM scores were similar across testing conditions, but suggested BPVS scores were higher for participants assessed online.	Online cognitive assessment does not necessarily lead to poor quality data for assessments.	Small and uneven sample size for each assessment condition in the study, which was a result of Williams Syndrome rarity and the restricted age inclusion criteria.
Athbah (2021) [21] Covid-19 impact on children with autism spectrum disorder and intellectual disability: Study in Saudi Arabia. Saudi Arabia.	To explore the impact of COVID-19 on children with autism spectrum disorder (ASD) and ID children from a parent’s point of view.	Quantitative – survey research. Sample − 217 parents (ASD and ID). Data collection – online survey. Data analysis – descriptive and inferential statistics. Timeline – not identified.	The impact of COVID-19 affected children with autism spectrum disorder and ID children greatly. There were no statistically significant differences attributed to the child’s gender, type of disability or parents’ education level.	The study recommends involving parents in their children’s rehabilitation sessions so that they can learn to train their children in the behaviours required in a time of public crises such as the Coronavirus pandemic.	Limited to those receiving services. May not be generalized.
Bailey et al. (2021) [22] COVID-19 impact on psychological outcomes of parents, siblings and children with intellectual disability: longitudinal before and during lockdown design. United Kingdom.	To explore the COVID-19 impact on psychological outcomes of parents, siblings and children with ID.	Quantitative – survey research. Sample – parents (pre-lockdown *n* = 294 and during/post-lockdown *n* = 103). Data collection – survey (online and paper). Data analysis – descriptive and inferential statistics. Timeline – April/July 2020.	During and shortly after the COVID-19 lockdown in the United Kingdom, well-being in families of children with ID (as reported by parents) was at similar levels compared with prior to the lockdown period.	Longitudinal research is required to understand the medium/long-term effects of COVID-19 on children with ID, their parental caregivers and their siblings.	Data may be most relevant to understanding the short-term impact of the COVID-19 lockdown and associated restrictions and not the medium and long-term effects when Covid-19 specific social policy support is withdrawn.
Baksh et al. (2021) [23] Understanding inequalities in COVID-19 outcomes following hospital admission for people with intellectual disability compared to the general population: A matched cohort study in the UK. United Kingdom.	To explore the hospital journey of patients with ID compared with the general population after they were admitted to hospital for COVID-19 during the first wave of the pandemic.	Quantitative – matched cohort study. Sample −506 persons with ID. Data collection – clinical records. Data analysis – descriptive and inferential statistics. Timeline – Feb/July 2020.	Symptoms such as loss of taste/smell were less frequently reported while altered consciousness and seizures were more common. Persons with ID admitted with higher respiratory rates and were more likely to require oxygen therapy. Persons with ID were less likely to receive non-invasive respiratory support, intubation and be admitted to ICU. 56% increased risk of dying from COVID-19 after they were hospitalized and were dying 1.44 times faster.	Significant disparities in healthcare between people with ID and the general population during the COVID-19 pandemic. This may have contributed to excess mortality and raises concern as to the possibility of systemic and professional bias and discrimination affecting treatment decisions in such conditions. Barriers to care will need to be overcome to ensure equality and the levelling up of services.	While data captured the accurate picture of acute clinical care at the time, the nature of clinical records can lead to missing or incomplete data. In addition, the use of combined group categories (particularly the heterogenous group ‘chronic neurological disorder’) limited the ability to explore the potential impact of specific diseases, while the reason for specific clinical decisions may not be clear. Data collected early in the pandemic and further data required to identify the long-term impact.
Bente et al. (2021) [24] The Dutch COVID-19 Contact Tracing App (the CoronaMelder): Usability Study. Netherlands.	To explore if the CoronaMelder app is user-friendly, understandable, reliable, credible, and inclusive?	Mixed methods – qualitative and survey research. Sample − 4 people with ID. Data collection – interviews and survey. Data analysis – descriptive statistics and interview data coded. Timeline – June/July 2020.	People with ID did not understand how the app operates or why there is a delay between exposure/increased risk and receiving a notification. The app provides complex information and lacks explanations, so users found it unclear as to what actions the app expects from them affecting adoption of the app and adherence.	The effectiveness of the app is a concern given that people with ID do not know how to use it. Staff are unprepared to support users with the app to the fullest extent during the pandemic (e.g. with key-sharing procedures) in addition to their other responsibilities.	Very small sample size in the study raises the question of whether the current findings can be generalized. Data collected very early in the pandemic and further data required to identify the long-term impact.
Bond et al. (2021) [25] COVID-19: experiences and contributions of learning disability nurses during the first wave of the pandemic. United Kingdom.	To explore the experiences and contributions of ID nurses during the first wave of the COVID-19 pandemic.	Qualitative – qualitative descriptive. Sample − 426 ID nurses. Data collection – reflective group discussions *via* video conferencing. Data analysis – thematic analysis. Timeline – Oct/Nov 2020.	Staff experienced organizational adjustments through adapting virtual service delivery, redeployment, expanding services and reducing social isolation. Staff had to focus on preserving health and well-being of service users and staff. Virtual transformation had challenges and advantages for virtual working, digital exclusion and innovations in communication.	Greater insight into how organizations and nurses managed the transformation of services during COVID-19. Nurses responded by adapting service delivery to become virtual, performing proactive risk assessments, ensuring physical health and well-being were maintained, and coming together as a team to ensure staff well-being did not deteriorate. Recognition of the unique contribution of ID nurses.	In some discussions, the group dynamics may have limited participants’ contributions. Participants were mostly working in the community not residential services. This limited the discussion and reflection on the experiences of ID nurses during the pandemic in inpatient settings.
Bullard et al. (2021) [26] Exploring the feasibility of collecting multimodal multi-person assessment data via distance in families affected by fragile X syndrome. United States of America and Canada.	To explore the feasibility of using telehealth procedures to collect multimodal behavioural and psychological assessment data from families.	Mixed method – survey research and qualitative. Sample − 19 mother child dyads. Data collection – online survey, interview, observations. Data analysis – descriptive. Timeline – not identified.	Telehealth procedures were successfully implemented across a wide range of technology platforms with limited difficulty, and we documented little missing data due to technology-related challenges. Mothers reported high satisfaction with participating *via* distance.	A wide range of services and assessments may be amenable to telehealth procedures. Telehealth may ease the burden of travel and eliminate the need for onsite assessments. Future work to collect behavioural and developmental measures.	The study was limited to data collection primarily on the part of a cognitively able adult and self-report measures also used. Online questionnaire may be limited to those with access and connectivity and literacy issues may affect response rate. Small sample size raises the question of whether the current findings can be generalized.
Carey et al. (2021) [27] Risk factors for excess all-cause mortality during the first wave of the COVID-19 pandemic in England: A retrospective cohort study of primary care data. United Kingdom.	To estimate the impact of risk factors on excess mortality in England during the first wave, compared with the impact on total mortality during 2015–2019.	Quantitative – retrospective cohort study. Sample − 22,453 (2020), 99,153 (2015–2019) patients with ID aged 30–104 years. Data collection – primary care data. Data analysis – descriptive and inferential statistics. Timeline – 18th March 2015 – 19th May 2020.	Risk factors where excess mortality was greatest and notably higher than usual included ID. The 2020 excess mortality ratio (EMR) (95% CI) for people with ID stood out at (8.54, 95% CI 5.99–12.18) more than double the 2015–9 usual mortality ratio (UMR) (95%CI) of 3.65, 95% CI 3.29–4.04).	Studying risk factors for excess mortality during the pandemic highlighted differences from studying cause-specific mortality. A novel methodology for evaluating impact by individual risk factor without requiring cause-specific mortality data.	Frequently an absence of data on potential confounding factors when data was recorded in the past.
Chemerynska et al. (2021) [28] What are the experiences of clinical psychologists working with people with intellectual disabilities during the COVID‐19 pandemic. United Kingdom.	To explore psychologists’ experiences of working with people with ID during the pandemic.	Qualitative – interpretative phenomenological analysis. Sample − 11 psychologists. Data collection - interviews. Data analysis – interpretative phenomenological analysis Timeline – March 2021.	Survive or thrive highlighted the challenges and successes clinical psychologists experienced while working during the pandemic. Left to Their Own Devices described psychologists’ experiences of their clients as forgotten within society.	The study demonstrates psychologists’ ability to adapt to extremely challenging circumstances, exposes the vulnerabilities of people with ID and highlights the gaps in service provision.	The small sample size.
Curtis et al. (2022) [29] Trends and clinical characteristics of 57.9 million COVID-19 vaccine recipients: A federated analysis of patients’ primary care records using OpenSAFELY. United Kingdom.	To describe trends and variation in vaccine coverage in different clinical and demographic groups in the first 100 days of the vaccine rollout.	Qualitative – cohort study. Sample − 1302 persons with ID. Data collection - electronic health records. Data analysis – descriptive and inferential statistics. Timeline – December 2020 - March 2021.	A total of 1190 (91.4%) of people with ID received a vaccine. Vaccination in the first 100 days of the vaccine rollout was significantly lower among people with learning disability.	Targeted activity may be needed to address lower vaccination coverage observed among certain such as ID. Live data monitoring is likely to help support those on the frontline making complex operational decisions around vaccine rollout.	The population, although extremely large, may not be fully representative of the full eligible population: it does not include individuals not registered with a general practice; or the 4% of patients registered at practices not using - electronic health records. Primary care records, while detailed and longitudinal, can be incomplete on certain patient characteristics.
Das-Munshi et al. (2021) [30] All-cause and cause-specific mortality in people with mental disorders and intellectual disabilities, before and during the COVID-19 pandemic: cohort study. United Kingdom.	To assess mortality risk prior to and during the COVID-19 pandemic over a range of mental disorder and ID diagnoses, and includes diagnoses (personality disorders, eating disorders and ID).	Quantitative – prospective study. Sample − 6045 persons with ID. Data collection – deaths from 2019 to 2020, used to assess standardised mortality ratios (SMRs) Data analysis – descriptive and sensitivity analysis. Timeline − 01 January 2019 − 31 December 2020.	Prior to COVID-19 all-cause SMRs across all psychiatric cohorts were more than double the general population. By the second quarter of 2020, all-cause SMRs increased further, with COVID-19 SMRs elevated across all conditions (notably: ID: SMR: 9.24). Increased SMRs were similar across ethnic groups.	People with and ID were at a greater risk of deaths relative to the general population before, during and after the first peak of COVID-19. Mortality from non-COVID-19/other causes was elevated before/during the pandemic, with higher COVID-19 mortality during the pandemic.	Not possible to assess mortality due to ‘natural’ or ‘unnatural’ causes, as the equivalent data unavailable. Ethnicity analysis limited by small numbers in some c groups.
Desroches et al. (2021) [5] Impact of COVID-19: Nursing challenges to meeting the care needs of people with developmental disabilities. United States of America.	To assess the challenges faced by nurses caring for persons with ID during the COVID-19 pandemic and how the challenges impact people with ID.	Quantitative – survey research. Sample − 556 ID nurses. Data collection - online survey. Data analysis – descriptive and inferential statistics, content analysis for open-ended questions. Timeline – April 2020.	Nurses reported being excluded from COVID-19 planning, and an absence of public health guidelines specific to persons with ID despite their high-risk status. Obtaining PPE and sanitizers and meeting social-behavioural care needs were the most highly ranked challenges. COVID-19 impacted nurses’ ability to maintain adequate staffing and perform essential aspects of care.	ID nurses must be involved in public health planning and policy development to ensure that basic care needs of persons with ID are met, and the disproportionate burden of COVID-19 in this vulnerable population is reduced.	Survey tool developed for the study. Data collected early in the pandemic.
Domagała-Zyśk (2021) [31] Attitudes of different age groups toward people with intellectual disability during the COVID-19 pandemic. Poland.	To explore the attitudes of adults towards people with ID in Poland.	Quantitative – survey research. Sample − 223 people. Data collection - online survey. Data analysis – descriptive and inferential statistics. Timeline – May 2020/January 2021.	The general attitudes were only slightly supportive and differed among people of different age groups. The youngest and the oldest generation expressed the most positive attitudes while the adults (35–60 yrs.) expressed the most negative attitude.	As the pandemic is spreading rapidly with no definitive solution, awareness to create more positive attitudes towards people with ID and recognising their needs is essential.	The study sample does not allow for generalizations. Only basic demographic data were collected, and little correlation analysis conducted.
Embregts et al. (2021) [32] Experiences of mothers caring for a child with an intellectual disability during the COVID‐19 pandemic in the Netherlands. Netherlands.	To explore the experiences and needs of parents caring for a child with ID during the first lockdown period in the Netherlands.	Qualitative – descriptive. Sample − 5 mothers. Data collection – semi-structured interviews Data analysis – Thematic analysis. Timeline – March/May 2020.	Mothers had an urge to protect their child’s well-being. They made things work, by handling the drastic changes in their family. Mothers valued their child’s and family’s place in the world, which focuses on the mothers’ experienced position in the world around them.	The study provides valuable insights into the experiences and needs of mothers caring for a child with ID during the COVID-19 pandemic.	The small sample size. Data collected early in the pandemic.
Embregts et al. (2022) [33] The experiences of psychologists working with people with intellectual disabilities during the COVID‐19 crisis. Netherlands.	To explore the experiences of psychologists working with people with ID during the initial stage of the COVID-19 lockdown in the Netherlands.	Qualitative – descriptive. Sample − 5 psychologists. Data collection - recorded 22 audio messages. Data analysis – thematic analysis. Timeline – focused on initial lockdown 15 March to 11 May 2020.	Three themes identified: (a) Working from home; (b) Adapting to the new reality; and (c) Advising and coaching support staff.	This study provides critical insights into the experiences of psychologists working with people with ID during the initial stage of the COVID-19 lockdown. These insights can help policymakers and practitioners to prepare for either a potential second wave of COVID-19 or a future pandemic.	The small sample size in the study raises the question of whether the current findings can be generalized.
Festen et al. (2021) [34] Determining frailty in people with intellectual disabilities in the COVID‐19 Pandemic. United States of America.	To compare the classification of individuals with ID into different frailty categories based on the Clinical Frailty Scale (CFS) and the well-studied ID-frailty index and to determine suitability of CFS for evaluation of frailty in individuals with ID during the COVID-19 pandemic	Quantitative – retrospective study. Sample −982 individuals with ID Data collection - observational healthy aging and ID (HA-ID) study. Data analysis – CFS and the ID-frailty index. Timeline – not identified.	63.7% classified as moderately frail (CFS score 6), but 92% not moderately frail according to the ID-frailty index. Furthermore, 20.3% classified as at least severely frail (CFS score 7–9), but 74.9% not severely frail according to the ID-frailty index. Overall, 74.9% would be incorrectly classified by the CFS as too frail to have a good probability of survival. The ID-frailty index predicts mortality better than the CFS in individuals with ID.	The CFS is not suitable to evaluate frailty in individuals with ID, with potential dramatic consequences for triage and decision-making during the COVID-19 pandemic. Strongly recommend using the ID-frailty index when assessing probability of survival for individuals with ID.	Time period not evident and frequently there is an absence of data recorded and under recognition of mild ID. Levels of ID not identified.
Fisher et al. (2022) [35] Social support as a mediator of stress and life satisfaction for people with intellectual or developmental disabilities during the COVID‐19 pandemic. North America, Asia, Europe.	To examined factors that predict stress level and life satisfaction among adults with ID during the COVID-19 pandemic and the role of social support.	Quantitative – survey research. Sample −181 adults with ID (or proxy). Data collection - online survey. Data analysis – descriptive and inferential statistics. Timeline – April 2020 until late-May 2020.	92.8% reported negative impacts from the pandemic, with 55.2% employed pre-pandemic reporting impacted employment, including job loss. The negative impact of the pandemic was a significant predictor of stress level; social support was related to reduced stress. Stress level and the negative impact of the pandemic were inversely related to life satisfaction; social support was positively related to life satisfaction. Social support partially mediated the association between stress level and life satisfaction.	Comprehensive services and social support systems are needed to combat the impact of the pandemic	First, although native language speakers provided translations, there is the possibility of translation and interpretation issues. Possible cultural differences or how countries responded to the pandemic socially and politically. Proxy respondents may have mispresented the experiences of individuals with ID. Although a global study, the majority of respondents lived in the United States, Germany or South Korea.
Flynn et al. (2021) [36] The experiences of adults with learning disabilities in the UK during the COVID-19 pandemic: qualitative results from Wave 1 of the Coronavirus and people with learning disabilities study. United Kingdom.	To present data about the experiences of adults with ID during the COVID-19 pandemic across the UK.	Mixed method – qualitative and survey research. Sample −609 adults with ID, 351 family carers/support staff. Data collection – interviews and online survey Data analysis – content analysis and descriptive statistics. Timeline - December 2020 – February 2021.	Social isolation was the most reported worry/negative for adults with ID, with other frequently reported worries/negatives including changes to/loss of routine; loss of support/services; and decreased health/wellbeing/fitness. A large proportion of participants indicated that nothing positive had happened because of COVID-19, but some positives were reported, including digital inclusion; more time spent with important people; improved health/well-being/fitness and, a slower pace of life.	Future pandemic planning must ensure that adults with ID are supported to maintain social contact with the people who matter to them and to support their health and well-being (including maintaining access to essential services and activities). Some adults with ID may benefit from additional support to improve their digital confidence and access. This may in turn enable them to maintain contact with family, friends and support services/activities.	Self-report measures also used. Online questionnaire may be limited to those with access and connectivity and literacy issues may affect response rate.
Flynn et al. (2021) [37] Health and social care access for adults with learning disabilities across the UK during the COVID-19 pandemic in 2020. United Kingdom.	To present data about access to health and social care services during the COVID19 pandemic for adults with ID across England, Northern Ireland, Scotland and Wales.	Mixed method – qualitative and survey research. Sample − 621 adults with ID, 378 family carers/support staff. Data collection - interviews and online survey Data analysis – content analysis and descriptive statistics. Timeline – March 2020 and February 2021	Access to and use of health and social care services significantly reduced for adults with ID across the UK during the COVID-19 pandemic between March 2020 and February 2021, with many people not receiving any services at all during that period. However, data suggest some variations between UK countries for some services.	Future pandemic planning must ensure that access to these essential services is not completely lost for adults with ID and their family carers, as it was in some cases during the COVID-19 pandemic in 2020.	Self-report measures also used. Online questionnaire may be limited to those with access and connectivity and literacy issues may affect response rate.
Friedman (2021) [38] The COVID-19 pandemic and quality of life outcomes of people with intellectual and developmental disabilities. United States of America.	To explore the impact of the COVID-19 pandemic on the quality-of-life outcomes of persons with ID.	Quantitative – survey research. Sample − 2284 people with ID. Data collection – structured survey interview using Personal Outcome Measures interview. Data analysis – descriptive and inferential analysis. Timeline − 2018 – 2020.	There were significant differences in the following quality of life outcomes of persons with ID between 2019 and 2020: continuity and security; interact with other members of the community; participate in the life of the community; intimate relationships and choose goals.	The COVID-19 pandemic negatively hindered the quality-of-life outcomes of people with ID. While the pandemic has been undoubtably hard on the ID community, in many ways it has simply intensified an underfunded and fractured ID service system. However, the ID service system evolves during and after the pandemic, it must be done in a way that prioritizes the quality of life of persons with ID and what is most important to them.	A convenience sample was used and there is a possibility of selection bias. This was an analysis of secondary data. therefore, additional variables or questions such as did the person contact COVID, could not be added.
Gacek and Krzywoszanski (2021) [39] Symptoms of anxiety and depression in students with developmental disabilities during COVID-19 lockdown in Poland. Poland.	To assess symptoms of anxiety and depression in persons with ID during COVID-19 lockdown.	Quantitative – survey research. Sample − 64 persons with ID and their parents or primary caregiver. Data collection – structured survey interview using GAD-7 and PHQ-8. Data analysis – descriptive and inferential statistics. Timeline – March - April 2020.	Over one third of the tested students reported mild or more severe symptoms of anxiety and depression, and girls were more affected than boys. The number of experienced lockdown inconveniences predicted the severity of depression symptoms in girls.	The high prevalence of symptoms of anxiety and depression in persons with ID indicates the need for screening studies and the provision of psychological help in situations such as the COVID-19 lockdown.	The small sample size in the study raises the question of generalizability. Baseline level of depression and anxiety symptoms before the pandemic occurred was not assessed so there is a possibility that other factors influenced the results.
Gale and Boland (2021) [40] COVID-19 deaths in a secondary mental health service. United Kingdom.	To describe the distribution of cases of COVID-19 recorded within a UK mental-health trust and, specifically, whether COVID-19 was reported more frequently for certain diagnostic groups.	Quantitative – retrospective study. Sample − 1181 patients of which 88 had ID. Data collection – record of Covid-19 cases. Data analysis – descriptive and inferential statistics. Timeline − 01 March − 31 October 2020, follow-up 31 January 2021.	Within the ID data there were 88 (8% of the patients), 12 (14%) died from Covid compared with 9% off the overall patients.	ID was the second most affected group after people with dementia. ID had better outcomes as 86% survived compared with people with dementia were only 39% survived.	The small sample size in the study raises the question of whether the current findings can be generalized.
Geuijen et al. (2021) [41] A qualitative investigation of support workers’ experiences of the impact of the COVID-19 pandemic on Dutch migrant families who have children with intellectual disabilities. Netherlands.	To explore the pandemic’s impact on Dutch migrant families who have children with ID, by interviewing these families’ support workers.	Qualitative – descriptive. Sample − 34 support workers providing data relevant to 27 Dutch migrant families who have children with ID Data collection – interviews. Data analysis – thematic analysis Timeline – May – June 2020.	The work of support workers during the COVID-19 pandemic did not go unnoticed in trying to maintain contact and build trust. The impact of COVID-19 upon migrant families who have children with ID was evident in increased burden, loss of relationships and fear of COVID-19.	The study demonstrates that support workers particularly struggled to stay in touch with migrant families who have children with ID during the COVID-19 pandemic. Therefore, support workers should tailor their support to the needs of migrant families.	The small sample size in the study raises the question of whether the current findings can be generalized. The findings reflect the early period of the pandemic. For this reason, longitudinal studies about the long-term consequences of the pandemic are needed.
Gil-Llario et al. (2021) [42] Sexual health in Spanish people with intellectual disability: the impact of the lockdown due to COVID-19. Spain.	To analyze the extent to which the sexual behaviour of people with ID (with and without a partner) was affected during the lockdown.	Quantitative – survey research. Sample − 73 people with ID. Data collection – questionnaire using structured survey interview in person or videoconference. Data analysis – descriptive and inferential statistics. Timeline – May – June 2020.	The lockdown increased the sexual appetite of a third of the sample (38%), especially the youngest participants. Sexual activity focused on autoeroticism and online behaviour, particularly sending nude images of oneself (88%) and viewing pornography (83.6%). Rates of sexual abuse during this period were relatively high (6.8%).	The sexual activity of people with ID was important during the lockdown, and they had to adapt to the circumstances of isolation in a similar way to the general population. Technological improvements in terms of devices and connection quality at home allowed their sexual behaviour to be reoriented, opening the door to new risks for the sexual health of people with ID.	The small sample size in the study raises the question of whether the current findings can be generalized. The findings reflect the early period of the pandemic. For this reason, longitudinal studies about the long-term consequences of the pandemic are needed.
Hatton et al. (2021) [43] The willingness of UK adults with intellectual disabilities to take COVID‐19 vaccines. United Kingdom.	To report factors associated with willingness to take the COVID-19 vaccine.	Quantitative – survey research. Sample − 621 adults with ID and 348 family carers or support workers. Data collection – structured interview (people with ID), survey (family/support workers). Data analysis – descriptive and inferential statistics. Timeline – December 2020 – February 2021.	87% of people with ID were willing to take a COVID-19 vaccine, willingness was associated with white ethnicity, having already had a flu vaccine, gaining information about COVID-19 and knowing social restrictions rules. 81.7% of carers reported their family member with ID would be willing to take a COVID-19 vaccine, with willingness associated with white ethnicity, having a health condition, having had a flu vaccine, being close to someone who had died due to COVID-19, and having shielded during COVID-19.	Reported willingness to take the COVID-19 vaccine is high among adults with ID in the UK, with factors associated with willingness having clear implications for public health policy and practice.	Self-report measures also used. Online questionnaire may be limited to those with access and connectivity and literacy issues may affect response rate. The small sample size in the study raises the question of whether the current findings can be generalized.
Heslop et al. (2021) [44] Deaths of people with intellectual disabilities: Analysis of deaths in England from COVID‐19 and other causes. United Kingdom.	To understand the circumstances leading to death from COVID-19 in people with ID.	Quantitative – retrospective. Sample − 200 deaths. Data collection – data from the learning [intellectual] disabilities mortality review (LeDeR). Data analysis – LeDeR programme methodology. Timeline − 02 March − 09 June 2020.	People with ID differed from the general population in their symptoms of COVID-19 and age at death. The overall quality of care was rated similar to other deaths of people with ID. Concerns were raised relating to recognizing acute deterioration and do not attempt cardio-pulmonary resuscitation decisions.	Service improvements are indicated in the ways in which people with ID encounter COVID-19 and experience the disease.	The numbers for some variables are small. Regional differences were not reported, or deaths from specific genetic conditions e.g. Down’s syndrome. Conducted at a time of considerable demand on health/care services, which may affect the quality and completeness. The LeDeR programme was established as a service improvement initiative, not a study.
Honingh et al. (2021) [45] Implications of COVID‐19 regulations for people with visual and intellectual disabilities: lessons to learn from visiting restrictions. Netherlands.	To examine how people with an intellectual and visual disability and their families experienced the period in which it was mandated not to have any physical contact.	Qualitative – qualitative. Sample − 14 people with ID and visual disability and 12 of their relatives. Data collection – interviews. Data analysis – thematic analysis. Timeline - June to August 2020.	Themes: (1) the instructed regulations of the sheltered care facilities and the government; (2) the relation with family and friends; and (3) the consequences of COVID-19 and the regulations. Both relatives and residents were understanding of the difficult situation, but also expressed criticism about the chosen regulations, the communication thereof, and the practical implementation.	Both groups have experienced the interruption of close contact as emotional and difficult. However, positive consequences of the restrictions due to COVID-19 were mentioned. The results provide a list of recommendations for sheltered care facilities	The small sample size in the study raises the question of whether the current findings can be generalized. The findings reflect the early period of the pandemic. For this reason, longitudinal studies about the long-term consequences of the pandemic are needed.
Hüls et al. (2021) [46] Medical vulnerability of individuals with Down syndrome to severe COVID-19-data from the Trisomy 21 Research Society and the UK ISARIC4C survey. United Kingdom.	To explore specific vulnerabilities, clinical presentation, and outcomes of COVID-19 in individuals with Down Syndrome (DS).	Quantitative – survey research. Sample − 1046 were analyzed and compared with patients with and without DS. Data collection - online survey. Data analysis – descriptive and inferential statistics. Timeline – April and October 2020.	The mean age of COVID-19 patients with DS was 29 years. Similar to the general population, the most frequent signs and symptoms of COVID-19 were fever, cough, and shortness of breath. Joint/muscle pain and vomiting or nausea were less frequent, whereas altered consciousness / confusion were more frequent. Risk factors for hospitalization and mortality were similar to the general population with the addition of congenital heart defects as a risk factor for hospitalization. Mortality rates showed a rapid increase from age 40 and were higher in patients with DS even after adjusting for known risk factors for COVID-19 mortality.	Leading signs/symptoms of COVID-19 and risk factors for severe disease course are similar to the general population. However, individuals with DS present significantly higher rates of medical complications and mortality, especially from age 40.	Not all of the COVID-19 cases were confirmed by COVID-19-specific laboratory tests.
Iadarola et al. (2022) [47] COVID-19 vaccine perceptions in New York State’s intellectual and developmental disabilities community. United States of America.	To explore COVID-19 vaccine perceptions in individuals with ID, their family members, and those who work with them, to inform a state-wide vaccine information and messaging project.	Quantitative – survey research. Sample – 825family or worked with persons with ID. Data collection – online survey. Data analysis – descriptive and inferential statistics. Timeline – 19th January – 9th February 2021.	Approximately 75% intended to or had received the vaccine. Greater vaccine hesitancy was reported in younger individuals and those making decisions on behalf of a person with ID. Concerns included side effects and the swiftness of vaccine development. Black and Hispanic participants had heightened concerns about being an experiment for the vaccine. Trusted sources of information included healthcare providers and family members.	Vaccine preferences in this New York State disability community sample align with national data. Identified concerns suggest the need for community education that addresses misperceptions. Age and race differences in perspectives highlight the need for tailored education, delivered by trusted messengers.	Access to online survey. Convenience sample used. Survey did not specify a definition of support so unclear when respondent indicate need or don’t need support.
Kaya and Sahin (2021) [48] I did not even receive even a phone call from any institution: experiences and recommendations related to disability during COVID-19. Turkey.	To explore the experiences of families who have children with disabilities in Turkey during the COVID-19 pandemic	Qualitative – phenomenological design. Sample − 10 parents. Data collection - interviews Data analysis – content analysis. Timeline – not identified.	Parents were anxious during quarantine regarding their child’s education, uncertainty and that their child would deteriorate. Families need psychological support, and serviced did not provided any support, help, or aid during the quarantine process.	The reality of who will take care of him/her if anything happens to me (parent) arose.	Limited sample size and phone interviews conducted, while appropriate interpersonal relationship may not have developed. Time of data collection not identified.
Kim et al. (2021) [49] Changes in life experiences of adults with intellectual disabilities in the COVID-19 pandemics in South Korea. South Korea.	To explore the experiences of adults with ID from their perspective during the COVID-19 pandemic in South Korea, where most community-based services were suspended.	Qualitative – qualitative. Sample − 15 adults with ID. Data collection - interviews Data analysis – thematic analysis. Timeline – July 6 and 21, 2020.	Changes evident in daily life, health behaviours, family relationships, social relationships, and social participation. But they also developed new ways of adapting and finding a new normal.	The findings offer valuable evidence of ways to develop and stabilize community-based services during a pandemic, with insights into the experiences of people with ID.	The small sample size in the study raises the question of whether the current findings can be generalized.
Kim et al. (2021) [50] A qualitative study on parents’ concerns about adult children with intellectual disabilities amid the COVID‐19 pandemic in South Korea. South Korea.	To understand parents’ concerns about their adult child with ID due to the restriction of community-based services amid the COVID-19 pandemic in South Korea.	Qualitative – qualitative. Sample − 19 parents Data collection - interviews Data analysis – thematic analysis. Timeline – July 2020.	Participants worried that their adult child was not aware of the seriousness of COVID-19, was more susceptible to the COVID-19 virus, could not recognize self-infection and could have fatal consequences of getting infected with COVID-19. They expected challenges in their adult child’s life (losing a daily routine, being isolated, regression in skills, becoming bored, lacking physical activities and increased behavioural challenges) but also experienced adjustments and hopes	The study demonstrated parents’ worry about their adult child becoming infected with COVID-19, highlighting the urgent need for community-based services to address psychosocial challenges during the pandemic.	The small sample size in the study raises the question of whether the current findings can be generalized.
Lake et al. (2021) [51] The wellbeing and mental health care experiences of adults with intellectual and developmental disabilities during COVID-19. Canada.	To explore the health and wellbeing of adults with ID, including supports that would be most helpful during the pandemic, from their perspective.	Qualitative – qualitative interpretive study. Sample − 9 adults with ID. Data collection – interviews. Data analysis – thematic analysis. Timeline – June and early August 2020.	Key aspects: the impact of the pandemic on daily life and wellbeing; a need for connection; and availability and access to mental health supports.	Participants described significant challenges to their health and wellbeing related to the pandemic and public health measures, but also demonstrated remarkable resilience. Findings highlight ways to support the wellbeing of adults with ID and how social determinants impact mental health.	The small sample size in the study raises the question of whether the current findings can be generalized.
Landes et al. (2021) [52] Risk factors associated with Covid-19 outcomes among people with intellectual and developmental disabilities receiving residential services. United States of America.	To identify associations between demographic characteristics, residential characteristics, and/or pre-existing health conditions and COVID-19 diagnosis and mortality for people with ID receiving residential support services.	Quantitative – cohort study. Sample − 543 individuals with ID. Data collection – AHRC data for people receiving residential support services in New York. Data analysis – descriptive and inferential statistical analysis. Timeline − 01 March − 01 October 2020.	Median age was 57, case rate was 16,759 per 100,000, mortality rate was 6446 per 100,000, and case-fatality rate was 38.5%. Increased age, DS, an increased number of residents and chronic kidney disease were associated with COVID-19 diagnosis. Heart disease was associated with COVID-19 mortality.	Similar to the general population, increased age and pre-existing health conditions were associated with COVID-19 outcomes for people with ID receiving residential support services. Number of residents was also associated with more severe COVID-19 outcomes and increased risk of COVID-19 diagnosis for people with DS.	COVID-19 positive status is based upon symptomatic testing without contact tracing. Limited to 5 boroughs of New York City and residential sample only.
Landes et al. (2021) [53] COVID-19 outcomes among people with intellectual and developmental disability in California: The importance of type of residence and skilled nursing care needs. United States of America.	To determine the impact of residential setting and level of skilled nursing care on COVID-19 outcomes for people receiving ID services, compared to those not receiving ID services.	Quantitative – cohort study. Sample − 354,640 people receiving ID services. Data collection - publicly available California data on COVID-19 outcomes for people with ID receiving services. Data analysis – descriptive and inferential statistical analyses. Timeline - May-October 2020.	Those receiving ID services had a 60% lower case rate, but 2.8 times higher case-fatality rate. COVID-19 outcomes varied significantly among Californians receiving ID services by type of residence and skilled nursing care needs: higher rates of diagnosis in settings with larger number of residents, higher case-fatality and mortality rates in settings that provided 24-hour skilled nursing care.	Diagnosis with COVID-19 among Californians receiving ID services appears to be related to the number of individuals within the residence, while adverse COVID-19 outcomes were associated with level of skilled nursing care. When data is available, future research should examine whether these relationships persist even when controlling for age and pre-existing conditions.	Data available on COVID-19 outcomes among people with ID is scarce. Comparisons of results may be limited by restricted definition.
Landes et al. (2021) [54] COVID-19 case-fatality disparities among people with intellectual and developmental disabilities: Evidence from 12 US jurisdictions. United States of America.	To compare COVID-19 case-fatality rates among people with ID in 11 states and the District of Columbia that are publicly reporting data	Quantitative – epidemiology case-fatality. Sample − 12 jurisdictions. Data collection – Data on ID COVID-19 outcomes for Arizona, California, Connecticut, District of Columbia, Illinois, Louisiana, Maryland, New Jersey, Oregon, Pennsylvania, Virginia, and Washington. Data analysis – descriptive and inferential statistical analyses. Timeline − 31 March − 13 April 2021.	Comparison of case-fatality rates between people with ID and their respective jurisdiction populations demonstrates that case-fatality rates were consistently higher for people with ID living in congregate residential settings (fifteen instances) and receiving 24/7 nursing services (two instances). Results were mixed for people with ID living in their own or a family home (eight instances).	People with ID, especially those living in residential settings, are experiencing higher case-fatality rates from COVID-19 than the general population across multiple US jurisdictions. Short-term and long-term public health interventions addressing COVID-19 risks will not be able to properly address the needs of people with ID until all states begin reporting COVID-19 outcomes for this population	The data from the 12 jurisdictions does not provide any data related to level of services provided within home settings, which could indicate level of health or personal needs. The data does not provide the age, sex, or racial-ethnic distribution of cases or deaths. Reporting is not standardized affecting the level of consistency of data used in this study.
Langdon et al. (2021) [55] Occupational stress, coping and wellbeing among registered psychologists working with people with intellectual disabilities during the COVID-19 pandemic in the United Kingdom. United Kingdom.	To characterise the changes at work experienced by psychologists working with people with ID during the pandemic and whether these changes, stressors and aspects of working life were associated with mental wellbeing and occupational stress.	Quantitative – cross-sectional survey. Sample − 94 psychologists. Data collection – online survey. Data analysis – descriptive and inferential statistics, thematic analysis of free text answers and triangulation. Timeline − 10 May to 10 June 2020.	Occupational stress, learning new roles, demands at home, and changes due to COVID-19 were associated with poorer mental wellbeing, while uncertainty about the role, a shortage of personal protective equipment, and poorer mental wellbeing were associated with occupational stress. Two main themes emerged during the thematic analysis: being human and being an employee.	The wellbeing and occupational stress of psychologists working with people with ID have been affected during the pandemic. It is of note that almost a quarter of our sample reported having been redeployed.	Self-report measures used. Online questionnaire may be limited to those with access and connectivity and literacy issues may affect response rate. The small sample size in the study raises the question of whether the current findings can be generalized.
Lee et al. (2021) [56] A qualitative exploration of the experiences of school nurses during COVID-19 pandemic as the frontline primary health care professionals. China.	To explore the experiences of school nurses during the COVID-19 pandemic in Hong Kong	Qualitative – qualitative descriptive study. Sample − 19 school nurses. Data collection - semi-structured interviews Data analysis – thematic analysis Timeline – May – June 2020.	I found it very challenging and difficult to stop our students with ID not touching their eyes and noses	Difficult for person with ID to understand restrictions.	General school nurses only one mention of ID. Population not specific to ID.
LoPorto and Spina (2021) [57] Risk perception and coping strategies among direct support professionals in the age of COVID-19. United States of America.	To explore direct support professionals’ perceptions of risk to COVID-19 infection while providing services to people with ID.	Quantitative – survey research. Sample − 486 direct support professionals. Data collection – online survey. Data analysis – descriptive and inferential statistics, thematic analysis of free text answers. Timeline – June – July 2020.	75.46% Believed they could become infected simply because of their working environment. 89.86% believed the people with ID were more at risk of becoming infected and 18.27% has already supported a person with the infection.	Staff worried all the time about contracting the infection. Staff had the proper PPE to do their jobs safely; and were given the tools, training, and information to reduce risk of infection at work	Descriptive statistics only. No identification of ethical approval. Unclear as to the process of coding open responses using content analysis and moving to thematic analysis of codes.
Lunsky et al. (2021) [58] Covid-19 positivity rates, hospitalizations and mortality of adults with and without intellectual and developmental disabilities in Ontario, Canada. Canada.	To describe population-level COVID-19 testing and positivity rates for adults with ID and sub-analysis for Down Syndrome.	Quantitative – retrospective cohort study. Sample − 96,013 adults with ID. Data collection - Ontario, Canada health administrative databases. Data analysis – descriptive and inferential statistical analysis. Timeline − 15 January 2020 to 10 January 2021.	Rates of adults who tested positive for COVID-19 were higher for adults with ID (19.35 per 1000) than for adults without ID (15.07 per 1000). Adults with Down syndrome had the highest rates (21.33 per 1000). Persons with ID were more likely to be in the highest morbidity category, had higher rates of several health conditions or disabilities that could exacerbate COVID-19 complications including asthma, diabetes, COPD, dementia, cerebral palsy, epilepsy and mental illness. Adults with ID were 2.23 times more likely to die and for adults under 55, those with ID were 16.77 times more likely to die. Down syndrome was 3.65 times more likely to be hospitalized.	ID group was more likely to be under 30 and less likely to be over 80. Regarding morbidity, people with ID were less likely to be in the lowest morbidity category (1.6% vs. 4.5%), and more likely to be in the highest morbidity category (20.6% vs. 9.0%). Hospitalization rates were higher for those with ID relative to those without ID overall, as well as in the two younger age groups (overall: 2.21 times higher).	Difficulty to recognizing mild ID and some not classified on the system and missed.
Lunsky et al. (2021) [59] Predictors of worker mental health in intellectual disability services during COVID‐19. Canada.	To describe the experience of ID service workers and predictors of emotional Distress.	Quantitative – survey research. Sample − 838 workers supporting adults with ID. Data collection - online survey. Data analysis – – descriptive and inferential statistical analysis. Timeline – July – Aug 2020.	One in four reported moderate to severe levels of clinical distress. Additionally, 34% met screening criteria for anxiety and 21% met screening criteria for depression. Those who reported feeling more stress at work now compared to pre-pandemic were 3.41 times more likely to report clinical distress. Those prescribed medication to help them manage their mental health were 1.95 times more likely to report significant clinical distress and those receiving therapy were twice as likely to report significant clinical distress.	Older workers (>45 years old) were more likely to report significant clinical distress compared to younger workers. One in four workers reported experiencing moderate to severe clinical distress, one in three met screening criteria for anxiety and one in five met criteria for depression.	The measure used for depression and anxiety were brief. Survey captures one moment in time and may not identify causal relationships between the variables studied, such as whether distress.
Lunsky et al. (2021) [60] Beliefs regarding COVID‐19 vaccines among Canadian workers in the intellectual disability sector prior to vaccine implementation. Canada.	To explore attitudes of workers towards COVID-19 vaccination prior to rollout, to inform strategies to promote vaccine uptake.	Quantitative – survey research. Sample − 3372 workers in ID services. Data collection – online survey. Data analysis – descriptive and inferential statistical analysis. Timeline – January – February 2021.	62% reported that they were ‘very likely’ to get a COVID-19 vaccine, and 20% reported that they were ‘somewhat likely’. Those under 50 were more likely to express vaccination non-intent compared with those over 50. Information was gained from public health from websites (85%) and television/radio news (58%) and participants trusted public health websites (72%) and physicians or other health care providers (45%) most.	Vaccination strategies need to address beliefs that the vaccine is unnecessary because of good health, trust in the vaccine due to fast development and fear of side effects. Concerns of becoming ill were lower in vaccine non-intent group. The most trusted sources of COVID-19 information were healthcare institutions (80%) and employers (50%).	Overall, a total of 40% missing data across all predictors of vaccination intent, with 4% to 11% of missing data per predictor.
Lunsky et al. (2021) [61] ‘The doctor will see you now’: Direct support professionals’ perspectives on supporting adults with intellectual and developmental disabilities accessing health care during COVID-19. Canada.	To describe direct support professionals’ experiences assisting adults with ID in accessing virtual and in-person health care during COVID-19.	Quantitative – survey research. Sample − 942 direct support workers. Data collection – online survey. Data analysis – descriptive statistics and content analysis to examine the open-text responses. Timeline – July – Aug 2020.	In person visits (24%) were difficult for clients to wear their mask, not touch surfaces. Concerns with physical environment/space were raised; it was challenging to manage physical distancing when appointment rooms were small or there were no waiting areas. Virtual visits were less common (22%) but seen as of benefit due to aggression and time issues and better to be ‘seen’ that phone consultations. Phone visits (58%) were seen as easy and efficient and preferred over waiting in crowded waiting rooms. Certain condition require physical visits and it is hard to advocate over the phone.	Challenges in supporting people tolerate masks and understand efforts needed to ensure compliance with safety protocols. While virtual visits have advantages challenges with technology and compatibility of platforms used were issues of concern. Phone visits were challenged by not seeing or being seen by the doctor.	Descriptive statistics only. Survey instrument not described. Non validated survey instrument. Unclear if content analysis framework was used.
Maggio et al. (2021) [62] Improving cognitive functions in adolescents with learning difficulties: A feasibility study on the potential use of telerehabilitation during Covid-19 pandemic in Italy. Italy.	To assess the efficacy of cognitive telerehabilitation in adolescents with ID to overcome the treatment problems related to the COVID-19 pandemic lockdown.	Quantitative – feasibility study. Sample − 24 people with ID. Data collection - All received cognitive treatment *via* a specific telehealth app to stimulate the cognitive skills related to learning. Treatment daily session lasting up to 60 min, five days a week for 4 weeks. Assessed by a neuropsychological at the beginning and at the end of the program. Data analysis – descriptive and inferential statistical analysis. Timeline – not identified.	Significant pre-post-treatment differences were observed in reaction time, rapidity and accuracy; distributed attention; selective attention capacity and processing speed ability; attentional shifting and sustained attention, as well as in memory.	Participants demonstrated good usability and motivation during the training. Telerehabilitation could be a valid tool for the rehabilitation of specific cognitive skills in adolescents with ID.	Small scale feasibility study and no control group. Timing during COVID not identified.
McCausland et al. (2021) [63] The impact of COVID‐19 on the social inclusion of older adults with an intellectual disability during the first wave of the pandemic in Ireland. Ireland.	Explore how social inclusion has impacted on a group of participants drawn from the ID Supplement to the Irish Longitudinal Study on Ageing.	Quantitative – national longitudinal study. Sample – persons with ID, 444 prior to COVID and 62 after lockdown. Data collection – structured interview. Data analysis – descriptive and inferential statistical analysis. Timeline - Sept 2019 – Sept 2020 (ceased during initial COVID outbreak).	No significant difference in family contact between the pre-lockdown and post-lockdown. No significant differences between the pre-lockdown and post-lockdown or rates of frequent contact with friends. No significant differences in the mean score for weekly social activities, significantly higher access to and use of technology was reported by the post-lockdown group.	No evidence of impact on contact with friends, which may have been maintained through increased use of technology. No evidence of impact on the number of social activities engaged in, which may suggest that this population have relatively fewer opportunities for social activities in general or that they adapted during lockdown to engage in new types of activities.	Timing of the data and focus longitudinal study on ageing with a refocus to analysis in the context of COVID. Type of technology not explored
McKenzie et al. (2021) [64] It’s been adapted rather than impacted’: A qualitative evaluation of the impact of Covid‐19 restrictions on the positive behavioural support of people with an intellectual disability and/or autism. United Kingdom.	To explore the experiences of social care staff regarding the provision of positive behavioural support to people with an ID during Covid.	Qualitative – qualitative. Sample − 19 staff. Data collection – interviews. Data analysis – thematic analysis. Timeline – April - May 2020.	It is important to maintain routines and structure. Staff attempted to sustain meaningful activities by replicating them within the home. The importance of social relationships for quality of life was recognized and staff explored ways in which these could be maintained as much as possible by use of technology but not a substitute for face-to-face.	‘It’s been adapted rather than impacted an increased focus on active support turning what might have been previously seen as mundane tasks into more meaningful activities and engaging more meaningfully with staff and peers:	The research team are involved in positive behavioural support training and may had had some prior contact with the participants, which may have affect their responses.
McNally Keehn et al. (2021) [65] Telehealth evaluation of pediatric neurodevelopmental disabilities during the COVID-19 pandemic: clinician and caregiver perspectives. United States of America.	To explore clinician and caregiver perspectives regarding telehealth neurodevelopmental evaluation.	Quantitative – survey research. Sample − 254 evaluations. Data collection - online survey. Data analysis – descriptive and inferential statistical analysis. Timeline – May – July 2020.	A clinical diagnosis was provided in 72% of evaluations and 65 cases diagnosis deferred. The clinician had to provide technical support to assist caregivers to gain access to the telehealth platform at the start of the evaluation (19%). Telehealth evaluation was adequate to address caregivers’ concerns (65%).	Technology barriers exist in the use of telehealth but not associated with ability to provided diagnosis. Telehealth saved three or more hours on travel and attendance at clinic for 30% of caregivers.	Newly developed tool. Demographic data not available for all participants.
Mosbah et al. (2021) [66] Effects of the COVID-19 pandemic and lockdown on the mental and physical health of adults with Prader-Willi syndrome. France.	Effects of the COVID-19 infection and lockdown on mental and physical health in Prader-Willi syndrome (PWS)	Quantitative – cross-sectional study. Sample − 85 people with PWS. Data collection – structured interviews. Data analysis – descriptive and inferential statistical analysis. Timeline − 15 April − 15 May 2020.	67.1% of patients reported a decrease in daily physical activity. Feeding behaviour improved for 35.4% and did not change for 43.9%. Weight loss occurred for 49%, stable for 23.7%, and 27.5% gained weight. 12.9% displayed a recrudescence of behavioural disorders which were more frequent.	Lockdown during the COVID-19 pandemic was associated with positive effects for most French adults with PWS, with weight loss probably associated with a more favourable environment during this period. Difficulties for caregivers were linked to exacerbations of anxiety and the ban on going outside	Proxy reports used with no measures of activity levels or food consumption. Parents’ sense of their children’s behaviour may have been heightened during the lockdown. Single study with small sample, which may not be of the general population of people with PWS. Formal ethical approval not identified.
Munir et al. (2021) [67] An analysis of families’ experiences with young children with intellectual and developmental disabilities (IDDS) during COVID-19 lockdown in Pakistan. Pakistan.	To explore parents’ perspectives of COVID-19’s impact.	Qualitative – qualitative. Sample − 176 parents. Data collection - interviews. Data analysis – qualitative data analysis (not specified). Timeline – July - December 2020	Concerns related to lockdown and affected behaviour, social and emotional status, environmental and cultural changes. Parents are facing financial challenges, stress issues, child caring burden, limited health facilities and inadequate government funding. To cope with these challenges parents, develop, healthy food, exercising, engaging in enjoyable activities, gaming, and meeting with relatives. On the other hand, a few parents also expressed the benefits of COVID-19 lockdown by spending more time with their children, which reinforces their family ties.	Parents of children with ID are confronted with significant challenges that increase their stress such as financial constraints, social lockdown, deteriorated health conditions of their children, reduced or no health services, closure of educational institutes, negative behavioural impacts, and slowed development. Still, a subset of parents also reported the positive side of lockdown, indicating that staying at home resulted in improved relationships.	Formal ethical approval not identified.
Mupaku et al. (2021) [68] Transitioning to adulthood from residential childcare during COVID‐19: Experiences of young people with intellectual disabilities and/or autism spectrum disorder in South Africa. South Africa.	To explore the impact of COVID-19 on their transitional journey	Qualitative – qualitative. Sample − 6 care leavers with ID and 3 caregivers. Data collection - interviews Data analysis – thematic analysis. Timeline – June 2020.	Lockdown created difficulties in implementing a different routine which disrupted the transition and led to a regression of independent living skills and increased frustration, anxiety and depression. Became withdrawn and isolated leading to depression. Caregivers had to be vigilant to the emotional due to disruption in established routines. Created unique opportunities to deepen meaningful relationships and spend quality time with their caregivers.	Support service for care leavers with ID should be essential services. Continual facilitation towards independence and personal well-being is required. Caregivers require training in interdependent living programmes.	Identifies 6 participants with ID but also that 2 were diagnosed with autism and four with ID. No clear identification if persons with autism also had ID. Small sample size.
Murray et al. (2021) [69] The impact of COVID‐19 restrictions in the United Kingdom on the positive behavioural support of people with an intellectual disability. United Kingdom	To rate the impact of COVID-19 on factors related to positive behavioural support (PBS)	Quantitative – survey research. Sample − 58 social care staff. Data collection – online survey Data analysis – descriptive statistics and content analysis for open responses. Timeline – April/May 2020.	Restricted activities and social contact scored negatively while staff creativity in finding solutions and the positive use of the extra time scored positively. 82.5% felt PBS learning helped them to some extent by helping staff to provide support in positive, constructive ways that improves quality of life; understand the functional nature of behaviours and use this understanding and other evidence to inform interventions.	PBS learning appeared to help staff cope with the negative impact of the restrictions. COVID-19 restrictions had a neutral or somewhat positive impact on all but two areas: the activities and quality of life of the person that the participant supp	Sample size was relatively small, and the study was based on self-report. Only staff views ascertained.
Naqvi et al. (2021) [70] COVID-19 pandemic impact on psychotropic prescribing for adults with intellectual disability: an observational study in English specialist community services. United Kingdom.	To understand the impact of the pandemic by comparing psychotropic prescribing patterns during lockdown.	Quantitative – retrospective cohort study. Sample − 210 people with ID. Data collection – Data analysis – descriptive and inferential statistics. Timeline – Jan 2020 – Jan 2021.	There was an overall increase in psychotropic prescribing during lockdown in urban as compared with rural settings (11 v. 2%). Antidepressants and mood stabilizers reduced in the rural setting. ADHD medication, benzodiazepines and antipsychotics all increased in both setting.	Marked reduction in face-to-face contacts and an increase in virtual and telephone consultations during the pandemic.	Focus on comparing two services (urban/rural) opportunity to report change in total population reviewed not provided. Deemed as not requiring ethical approval but access to data and under GDPR an application for chairs approval may be warranted.
Navas et al. (2021) [71] Impact of COVID-19 on the burden of care of families of people with intellectual and developmental disabilities. Spain.	To explore the impact of COVID-19 and the response measures applied during lockdown on people with ID.	Quantitative – survey research. Sample − 582 individuals with ID. Data collection – online survey Data analysis – descriptive and inferential statistics and content analysis of open questions. Timeline – June 2020.	89.5 % of participants reported receiving information about COVID and 81% stating the information was easy to understand. 82.1 received support during the lockdown. 91.1 % reported an impact on social relationships. 51.5 % reported providing support to others during lockdown (family, roommates, friends).	Those living in specific settings had fewer natural supports, and those living with their family relied heavily on the family to address their needs because of service closure.	Those with sever to profound ID may not be represented in this study. Those that may have difficulties accessing technology or have communication problems may have been unable to participate.
Navas et al. (2021) [72] Supports for people with intellectual and developmental disabilities during the COVID-19 pandemic from their own perspective. Spain	To analyse the impact that COVID-19 and the response measures implemented by the Spanish Government.	Quantitative – survey research. Sample − 323 family members. Data collection - online survey. Data analysis – descriptive and statistics and thematic analysis of open-ended questions. Timeline – June 2020.	66.3% of families reported stress and anxiety. Excessive care burden and the difficulties faced to reconcile family and working life were the main reasons for this increase in stress and anxiety. 51.8% reported to have found difficulties to meet the support needs that the person required in everyday life. 74.3% stopped receiving a service, which had a negative emotional consequence (behaviour, loss of skills, sadness).	Families of those with higher support needs experiencing poorer outcomes. Changes experienced during lockdown vary according to service type. Families’ concerns were uncertainty regarding continuity of support and services, how the person will adapt and coexistence with the virus, the effects on physical and emotional wellbeing, and worry about the impact on employment.	Does not identify open ended questions or how many questions of responses. People who had limited access to technology may not be represented. Relatives’ perspective only.
Oudshoorn et al. (2021) [73] Experiences of therapists conducting psychological assessments and video conferencing therapy sessions with people with mild intellectual disabilities during the COVID-19 pandemic. Netherlands.	To explore therapists’ experiences of using video conferencing during the initial lockdown period.	Qualitative - phenomenological study. Sample − 7 therapists. Data collection - self-recording on smartphone (*n* = 5) or written experience (*n* = 2). Data analysis – thematic analysis. Timeline – not specified but focused on lockdown period of 16th March to 15th May.	Needed to support the setup of accounts (how to install, create an account and password and activate new app). Had to pay attention to the impact on service users and focus on how coping strategies were directed towards helpful ways of performing activities at home. Technology accessibility and unstable internet connections were issues. Service user often late or took part while doing other chores, easily distracted (checking phone) affecting participation and engagement. Unforeseen advantage was that some service users were more relaxed at home, resulting in an increased frankness in their discussions.	Therapists experienced that their own digital skills were insufficient and had to spend time and effort learning the necessary skills. Send materials to service users prior to session (workbook, paints, questionnaire, clay). Hesitant to use video conferencing for therapy with complex families, due to difficulties in observing the interpersonal interactions between family members. Participants experienced notable differences when comparing working with and without the support of staff or parents during assessments and therapy sessions	Small sample size. Data collection method may be limited in-depth of experience that could be gained.
Parchomiuk (2021) [74] Care and rehabilitation institutions for people with intellectual disabilities during the COVID-19 pandemic: Polish experiences. Poland.	To determine what changes took place in the rehabilitation and care institutions during the pandemic.	Quantitative – survey research. Sample − 148 institutions. Data collection – online survey. Data analysis – descriptive and inferential statistical analysis. Timeline – not specified.	Infection prevention and control measures were implemented. Difficulty in adhering to restrictions such as wearing a mask due to sensory, dexterity issues and high levels of anxiety created. Remote support, classes and meetings were facilitated but there were cases where, due to lack of competencies people could not use equipment that would allow them to participate. Burden on staff working in a different rhythm, with additional duties, adapting to new conditions, and use other work tools.	There is a need for financial resources for psychological support and technology use. Most experienced a decrease in mood, feeling of sadness, a sense of loneliness, boredom and inactivity. Risks associated with the loss of competencies, social exclusion, and isolation and predict an increase in the risk of mental and physical health conditions due to negative emotional experiences and difficulties in accessing therapy and specialist counselling.	Data obtained from people in managerial positions, and May be inclined to present their institution favourably. Low response rate for the four groups (2.38, 6.1, 6.95 and 17.6%) and overall response rate (8.9%).
Patel et al. (2021) [75] The experiences of carers of adults with intellectual disabilities during the first COVID‐19 lockdown period. United Kingdom.	To gain insight into parents of adults with ID coping during lockdown.	Qualitative – qualitative Sample −8 parents. Data collection – interviews. Data analysis – reflexive iterative process. Timeline – June – July 2020.	Felt powerless, not in control and not knowing what was going to happen. For some social isolation it’s just that everything else has shut down that makes it difficult. A difficulty for families was routine, maintaining routine and creating a new one. Services could be seen as not been supportive, but this relates to communication and the feeling of been left alone to cope. Parents felt exhaustion and that they had no time to relax.	The use of technology assisted social networking and service provision. Both positive and negative effects are experienced where the absence of services and isolation created anxiety and stress it also created opportunities to connect and communicate within families and take a break by stopping and deciding what activity to do. Caring impacts on carers physical and mental health.	No identification of qualitative philosophy underpinning study. Small sample size, randomly selected from expression of interest from previous survey (31 out of 51) no justification for exclusion of families who expressed an interest to participate but were not invited.
Paulauskaite et al. (2021) [76] My son can’t socially distance or wear a mask: how families of preschool children with severe developmental delays and challenging behavior experienced the COVID-19 pandemic. United Kingdom	To explore the experiences of families of young children with moderate to severe ID and challenging behaviour.	Quantitative – survey research. Sample − 88 parents. Data collection – online survey. Data analysis – descriptive statistics and content analysis of free text responses. Timeline – May – July 2020.	88% had to manage additional mental health problems, 71.6% loneliness, 56% lack of support from family and friends and increased family tensions. 15.6% of respondents reported increased substance misuse and gambling. 90.9% of parents reported difficulties maintaining adequate support for their child and abrupt disruption of access to usual support from health services (76%), education (90.9%), social care, and voluntary sectors (71.7%). Skills gained had dissipated during lockdown but fearful of resuming services during COVID.	Parents reported a lack of relevant information to assist them in explaining the changes in routine to the child. Parents experienced difficulties in following government requirements on social distancing, self-isolation and/or shielding and implementing infection control measures at home. Parents were dissatisfied with the use of video and telephone for assessments. E-mail and text messaging were preferred methods of keeping in contact with services.	Survey instrument developed but no validity testing.
Peacock-Brennan et al. (2021) [77] The experience of COVID-19 “lockdown” for people with a learning disability: results from surveys in Jersey and Guernsey. United Kingdom.	A service evaluation that gathered feedback from people with ID on their experience of lockdown	Quantitative – survey research. Sample − 96 people with ID. Data collection – accessible survey. Data analysis – descriptive statistics and thematic analysis. Timeline –	Most respondents reported feeling happy (66%), safe (68%), calm (51%). A minority of respondents reported “negative” emotions, feeling worried (34%), lonely (17%). Respondents valued spending more time with family, as well as contact with services, either directly or remotely using technology. Some people valued social distance and removal of social pressure, which helped them feel safer than at other times.	Most respondents felt safe, calm and happy and valued support from services. distance and disconnection from friends and family was one of the factors that people missed most.	Sample consists of those in receipt of service. Experience of islanders may be different from other jurisdictions as COVID cases on the islands were low.
Power et al. (2021) [78] ‘Reflecting or frozen?’ The impact of Covid-19 on art therapists working with people with a learning disability. United Kingdom.	To capture the experiences of art therapists and describe the barriers and facilitators for online art therapy with people with ID.	Qualitative – qualitative. Sample − 105 art therapists. Data collection - Six 60-minute Zoom sessions with handwritten record of each session. Data analysis – thematic analysis Timeline – October 2020.	There was a perception of a changed reality. Some clients required carer assistance to access the technology from home, whilst others lacked internet-enabled phones, which prevented access to tele-therapy. Experienced a loss of professional identity due to the sudden cessation of face-to-face clinical practice and the rapid introduction of online working.	The pandemic was an opportunity to reflect by pushing the pause button. Experienced job role changes. Increased reliance on communication with carers to access clients who were non-verbal or could not use computers, tablets or phones. Inter-personal and sensory aspects of the therapeutic relationship, which cannot easily translate across a computer screen.	Record sessions rather than handwritten record would have enhanced data collection.
Purrington et al. (2021) [79] Exploring maintaining gains following therapy during the coronavirus pandemic with adults with an intellectual disability. United Kingdom.	To explore service user experiences of maintaining gains following therapy within the context of the Covid-19	Mixed-method – survey research and qualitative. Sample − 9 clients with ID. Data collection – survey and interviews. Data analysis – descriptive and inferential statistics and framework analysis of qualitative data. Timeline – February 2021.	Following a mean of 10 sessions of therapy, service users reported reduced PTOS-ID II (psychological therapy outcome scale) distress scores and an increase in well-being scores. Service users reported greater PTOS-ID II distress and reduction in well-being scores at follow-up as compared to post-therapy scores. Service users reported enjoying talking and receiving help, feeling listened to, understood and contained.	Therapy had a moderate positive impact on service users’ PTOS-ID II distress and well-being scores and that follow-up distress scores demonstrate a mild-moderate regression with follow-up well-being scores demonstrating a mild regression. Therapy helped during the pandemic with service users reporting being able to cope with problems more easily, worrying less and dwelling on difficulties less.	Small sample size.
Rauf et al. (2021) [80] COVID-19-related prescribing challenge in intellectual disability. United Kingdom.	To identify if COVID-19 restrictions were associated with a change in consultations with psychiatrists and prescription of psychotropic medication within a community ID and/or autism spectrum disorder service.	Quantitative – observational study. Sample - *n* = 360 CAMHS ID consultations. Data collection - retrospective and prospective data collection before and during lockdown. Data analysis – descriptive and inferential statistical analysis. Timeline – January – June 2020.	During the lockdown period consultations increased by 19 per week and medication interventions increased by two per week. Multidisciplinary team input increased from 0.17 to 0.71 per week for children and from 5.7 to 6.5 per week for adults. Hypnotics and benzodiazepines were the most commonly prescribed psychotropic medications during the lockdown period.	COVID-19-related lockdown resulted in an increase in medication interventions, total consultations and involvement of multidisciplinary teams to manage mental health and behavioural issues in people with ID and/or ASD.	Mixed sample ID and/or ASD and persons with ASD may or may not have ID. All consultations were considered rather than the same patients compared in pre-lockdown and lockdown periods.
Rawlings et al. (2021) [81] Telephone-delivered compassion-focused therapy for adults with intellectual disabilities: a case series. United Kingdom.	To explore the feasibility, acceptability and preliminary effectiveness of delivering psychological therapy remotely to adults with ID.	Quantitative − survey research.Sample − 6 persons with ID. Data collection – survey. Data analysis – descriptive and inferential statistics. Timeline – June – Sept 2020.	A reduction was observed at post-therapy in distress and risk. No difference was reported in psychological well-being. All six clients reported finding the programme ‘helpful’ and enjoyed focusing on compassion.	Most clients found the intervention helpful, enjoyable and were pleased that they received telephone-delivered psychological therapy.	Small sample size. Differences in sessions completed by participants.
Rawlings et al. (2021) [82] Exploring how to deliver videoconference-mediated psychological therapy to adults with an intellectual disability during the coronavirus pandemic. United Kingdom.	To explore the accessibility and prospective acceptability of providing telephone and videoconference-mediated psychological interventions in individuals with ID.	Quantitative – survey research. Sample − 7 persons with ID. Data collection – survey via structured interview over the phone. Data analysis – descriptive statistics and analysis of open questions. Timeline – May 2020.	Most of the clients were unable to engage in video-conference therapy and therefore, only suitable for phone therapy. Providing clients with a photo of the therapist may also help to build alliances with clients. Clients reported at times having issues with the sound quality.	Images can be easier to process compared to words, which are more abstract in nature. Client often needed their carer on the phone for therapy to help them understand what is being discussed, posing confidentiality and engagement issues.	Small sample size.
Redquest et al. (2021) [83] Exploring the experiences of siblings of adults with intellectual/developmental disabilities during the COVID‐19 pandemic. Canada.	To identify helpful resources for siblings of persons with ID during COVID-19.	Quantitative – survey research. Sample − 91 siblings. Data collection - online survey. Data analysis – descriptive and inferential statistics and content analysis of open-ended questions. Timeline – May – July 2020.	Siblings are concerned about the health and well‐being of their brother/sister. The most common concern related to disruption of their siblings routine and activities. Support provided was categorized as provision of practical and emotional support, financial support, and informational support.	Siblings are providing key support to their siblings with ID and need support. Siblings should be included in efforts to disseminate resources, gain feedback and in mental wellness support.	Recruitment via social media and those with access issues may not be represented. 95% female sample.
Rogers et al. (2021) [84] The experiences of mothers of children and young people with intellectual disabilities during the first COVID‐19 lockdown period. United Kingdom.	To gain insight into the ways mothers of children with ID coped during the first 2020 lockdown.	Qualitative – qualitative. Sample − 8 mothers. Data collection - interviews. Data analysis – thematic analysis. Timeline – May – June 2020.	Lockdown restrictions put pressure on carers to develop new ways of managing caring responsibilities. Mothers reported difficulty in providing the level of support offered by external resources prior to lockdown. The burden of care, for some, impacted on their own mental health. The elimination of many daily pressures resulted in a reduction in challenging behaviours and improvements in mood, sleep, seizures, obsessive and compulsive routines, speech, and a number of children appeared more relaxed.	While mothers experienced increased burden and stress there were positive impacts of lockdown on children’s well-being and behaviour. Resilience was developed ads mothers developed effective coping strategies to combat depression, anxiety and feeling powerless by developing exercise routines or hobbies.	Relatively affluent sample. Small sample size.
Rosencrans et al. (2021) [85] The impact of the COVID-19 pandemic on the health, wellbeing, and access to services of people with intellectual and developmental disabilities. United States of America and Chile.	To explore mental health problems and services in individuals with ID during the pandemic	Quantitative – survey research. Sample − 468 people with ID. Data collection - online survey. Data analysis – descriptive and inferential statistics. Timeline – July – October 2020.	19 % not receiving services, 30% difficulty in accessing services and 44 % services had changed since COVID-19. 9% increased health issue, 15% difficulties in accessing medical treatment and 3% difficulty in accessing medicine. 29 % felt scared to go to the hospital/doctor if they were sick. 71% spending more time on screens and devices. 42 % accessing mental health therapy since pandemic began and experiencing more mental health problems with Chile reporting a 51.6 % increase in mental health problems.	Individuals with ID are vulnerable to changes associated with the COVID-19 pandemic given an increased likelihood of health concerns, low socioeconomic status, and difficulty accessing services.	Methodologies different for the US and Chilean studies.
Rousseau et al. (2021) [86] Clinical characteristics of COVID-19 infection in polyhandicapped persons in France. France.	To describe the characteristics of this infection among individuals with polyhandicap.	Quantitative – survey research. Sample −98 persons with polyhandicap. Data collection – survey. Data analysis – descriptive and inferential statistics. Timeline − 01 April − 01 July 2020.	Fever was the most constant symptom (68.4%). The most frequent respiratory symptoms were dyspnea (20.6%), hypoxemia (28.9%), and bronchial congestion (21.6%).	Clinicians should be aware that COVID-19 symptoms are often extra-respiratory signs, mostly digestive and neurologic, which may help in the earlier identification of COVID-19 infection.	Specific group with ID defined by the combination of motor deficiency, profound mental retardation, and age at onset of cerebral lesion younger than 6 years
Ryzhikov (2021) [87] Virtual therapeutic horticulture – A social wellness program for adults with intellectual and developmental disabilities. United States of America.	To assess the feasibility of delivering a therapeutic horticulture program *via* a virtual platform with adults with ID.	Quantitative – survey research. Sample −39 adults with ID. Data collection – survey. Data analysis – Timeline – not specified.	The program was very well received (97%) and rated highly. 85% enjoyed receiving and working on the activity kits. 80% enjoyed participating via computer. 97% indicated participating on the computer helped them stay connected to their friends/peers.	If had a choice, they would rather in-person sessions. Participants actively engaged and responded positively to the program. A virtual platform may be a feasible method to deliver a therapeutic program and may provide an opportunity for enhanced social wellness.	Reliance on the assistance of family and staff to complete the questionnaire. Ethics not identified.
Salzner et al. (2021) [88] Deaf residents with intellectual disabilities during the first Covid-19 associated lockdown. Austria.	To investigate changes in mood and frequency and severity of aggressive behaviour of people who are deaf/hard of hearing and have ID 8 weeks pre and post the first lockdown.	Quantitative – cohort observational study. Sample − 38 persons with ID. Data collection - staff observation of recorded on aggression scale. Data analysis – descriptive and inferential statistics. Timeline – Jan – May 2020.	A small significant improvement in the mood ratings occurred between t1 and t2. Severity of aggressive behaviour measures changed significantly between t1 and t2. Physical aggression toward others showed the highest incidence rate both at t1 and t2.	With proper communicative support, individuals can cope effectively with significant restrictions imposed by a pandemic/lockdown.	Limited in statistical tests due to sample size.
Sanchez-Larsen et al. (2021) [89] COVID-19 prevalence and mortality in people with epilepsy: A nation-wide multicenter study. Spain.	To assess the prevalence, severity, and mortality of COVID-19 in people with epilepsy.	Quantitative – retrospective, observational, multicenter study. Sample - 397people with ID. Data collection – medical records. Data analysis – descriptive and inferential statistics. Timeline – Jan – Dec 2020.	31tested positive for COVID-19. Persons with ID were more likely to contract COVID-19. Having a severe form of COVID-19 was more likely to occur with ID and in long-term care facilities.	ID acted as a risk factor for contracting COVID-19 and for having a severe course of the disease, though it was not associated with higher mortality.	Mixed sample group only 14.4% with ID.
Scheffers et al. (2021) [90] Assessing the quality of support and discovering sources of resilience during COVID-19 measures in people with intellectual disabilities by professional carers. Netherlands.	How professionals support and rate the quality support compared to before COVID-19 measures and identify sources of resilience.	Quantitative – survey research. Sample − 290 professional carers. Data collection - online survey. Data analysis – descriptive and inferential statistics. Timeline − 21 April − 08 June 2020.	Professional carers applied diverse and distal methods to maintain contact with people with ID during the COVID-19 measures. Professional carers reported a significant decrease in the quality of contact with clients with ID, but overall high levels of resilience in clients. The 3 most frequently reported source of resilience of clients was ‘positive thinking (85.9 %), other relationships (82.8 %), and intimate relationships (68.1%).	Online methods of communication are possibly insufficient for professionals to cover all needs of people with ID. Professionals should be aware of stress but also of resilience of people with ID.	Not all questionnaires were completed, and missing data not addressed in analysis.
Schott et al. (2021) [91] COVID-19 risk: Adult Medicaid beneficiaries with autism, intellectual disability, and mental health conditions. United states of America.	To identify COVID-19 risk.	Quantitative – cohort study. Sample − 615,607 persons with ID/ID and ASD. Data collection - Medicaid Analytic eXtract (MAX) data. Data analysis – descriptive and inferential statistics. Timeline – August 2020.	Individuals with ID had higher prevalence of avoidable hospitalizations which was over twice as high compared to the general Medicaid sample. Individuals with ID had nearly two-three times as many comorbidities as the general Medicaid sample.	High risk of COVID-19 among adults with ID should be recognised and these groups should be prioritised for vaccine outreach.	Individuals for whom conditions were not coded could be missing from the data and lead to an underestimate of risk factors. Ethics not identified.
Sheehan et al. (2021) [92] Effects of the COVID-19 pandemic on mental healthcare and services: Results of a UK survey of front-line staff working with people with intellectual disability and/or autism. United Kingdom.	To explore the challenges and innovations reported by staff working in services for people with ID.	Quantitative – survey research. Sample − 648 staff. Data collection - online survey. Data analysis – descriptive and inferential statistics and content analysis for open ended questions. Timeline – April – May 2020.	staff identified more problems with implementing precautions designed to minimise transmission of infection, including lack of personal protective equipment, difficulty putting infection control measures into place and problems resulting from lack of access to testing.	Staff had to adapt quickly to new ways of working, using new technologies without adequate training or support. Issues in not having necessary tools or equipment to facilitate remote working was evident.	Secondary analysis of data from a larger survey. Large number of questions which lead to 28.2% been discarded as incomplete the remaining were included as had at least one valid response in each of the main section which also may indicate not fully completed.
Suarez-Balcazar et al. (2021) [93] Impact of Covid-19 on the mental health and well-being of latinx caregivers of children with intellectual and developmental disabilities. United States of America.	To explore the impact of the pandemic on the mental health and well-being of Latinx caregivers of children with ID	Mixed methods – survey research and qualitative. Sample − 37 caregiver-child dyads Data collection – survey and interview. Data analysis – descriptive and inferential statistics and thematic analysis. Timeline – July 2020 – March 2021.	38% of families reported having low or very low food security. 54% of children were able to maintain access to two of the three service categories (i.e. online special education, routine therapies, and ID related services). Common stressors for caregivers included concerns about family finances and sustained employment, lack of support with childcare, and worries about family members getting sick. Concerns about children’s mental health mostly stemmed from lack of social interaction due to the pandemic lockdown. The pandemic presented opportunities for bonding, spending more time together as a family, sharing meals and playing board games.	Greater perceived social support was significantly correlated with greater energy/fatigue scores and lower depressive symptoms scores. The pandemic presented both concerns and challenges, as well as opportunities for families. The break in routine meant that mornings were less stressful, and children were able to sleep in and get more rest.	Findings may be limited in their generalizability to the larger population of Latinx families and non Latinx families. Small sample size for statistical analysis.
Vereijken et al. (2022) [94] Homeward bound: Exploring the motives of mothers who brought their offspring with intellectual disabilities home from residential settings during the COVID‐19 pandemic. Netherlands.	To explored why mothers decided to bring their offspring home.	Quantitative – interpretative phenomenological approach. Sample − 7 mothers of adults with ID. Data collection - interviews. Data analysis – interpretative phenomenological analysis. Timeline – March – June 2020.	All mothers were concerns for their offspring’s wellbeing due to uncertainty that their offspring would be safe in the residential facility. Their initial impulse to take their child home as being based on an instinctive desire to have their children with them during a time of crisis. Parents reflected on their role and the fact that their offspring had grown into adults, were making their own decisions and difficulty in setting boundaries.	COVID-19 outbreak prompted some mothers to make formal arrangements relating to the future care of their offspring.	Sample size and clients represented were female (*n* = 6) male (*n* = 1).
Wieting et al. (2021) [95] Behavioural change in Prader–Willi syndrome during COVID-19 pandemic. Germany.	To assess the impact of the restrictions associated with the COVID-19 pandemic on the mental health of people with Prader–Willi syndrome.	Quantitative – survey research. Sample − 108 caregivers. Data collection – online survey. Data analysis – descriptive and inferential statistics. Timeline – August 2020.	Restrictions in the provision of care and employment/daytime activity were reported frequently. 51.7% increase in temper outbursts, 46.1% increase in conflicts with other people and 55.0% increase in irritability. 43.8% increase in depressed mood, 38.2% increase in anxiety and 33.7% increase in social withdrawal. Increased daytime sleepiness (40.4%) and an increased desire to eat (39.3%). Obsessive–compulsive behaviour increased 33.0%, skin picking 50.6%, psychotic experiences 26.4% and suicidal thoughts 5.6%.	There were sufficient resources (e.g. masks, disinfectants) to implement protective measure. COVID-19 pandemic has had a significant effect on the mental health, evidenced by an increase in behaviours such as temper outbursts, food-seeking, and irritability. There is a need for specialized care and families are in special need of support.	Indicates a sample of 89 in the article also raising confusion as to the actual sample size.
Williamson et al. (2021) [96] Risks of Covid-19 hospital admission and death for people with learning disability: Population based cohort study using the OpenSAFELY platform. United Kingdom	To assess the association between ID and risk of hospital admission and death from Covid-19	Quantitative – cohort study. Sample − 90,307 were on the ID register. Data collection - inpatient activity datasets. Data analysis – descriptive and inferential statistics. Timeline – March 2020 – February 2021.	538 COVID-19 related hospital admission and 222 related deaths. Hazard ratios were 5.3 for COVID-19 related hospital admission and 8.2 for COVID-19 related death. Associations were stronger among those classified as having severe to profound ID, and among those in residential care. DS and cerebral palsy were associated with increased hazards.	People with ID have markedly increased risks of hospital admission and death from Covid-19. Prompt access to Covid-19 testing and healthcare is warranted for this vulnerable group, and prioritization for Covid-19 vaccination and other targeted preventive measures should be considered.	Cerebral palsy sample may or may not have ID.
Wolstencroft et al. (2021) [97] We have been in lockdown since he was born’: A mixed methods exploration of the experiences of families caring for children with intellectual disability during the COVID-19 pandemic in the UK. United Kingdom.	To explore the experiences of parents caring for children with ID during lockdown.	Qualitative – qualitative. Sample − 23 parents. Data collection - interviews. Data analysis – thematic analysis. Timeline – July 2020.	Social distancing had been part of family’s daily life before the pandemic. Parents had to manage an increase in behavioural problems uncertainties in coordinating care and worries about explaining COVID-19. Demands placed on families due to fewer resources, such as respite care, school, social care and their support networks and changes in routine. Children strongly impacted by not being able to see friends or extended family and missed both physical and emotional contact with others. For some a positive impact on mental health and well-being as less stress and anxieties and more relaxed environment.	Parents had difficulty adjusting to the pandemic as challenges arose in managing pre-existing challenges. There were unexpected benefits and unanticipated challenges causing mixed emotions. Personalized supports are essential for their families to get through the lockdown. Many children had difficulty upholding social distancing measures thus parents avoided certain situations to keep their children safe. Life skills and social skills had regressed due to the lack of contact with others. Telehealth is not an option for all some need face-to-face and other require high level of support to engage.	Part of a mixed methods study and provided details on mixed methods but focus is on the qualitative so reporting quantitative measure unnecessary and lead to less depth and clarity of qualitative approach. Only mother in the sample.
Wos et al. (2021) [98] Remote support for adults with intellectual disability during COVID‐19: From a caregiver’s perspective. Poland.	To explore the experiences of parents of adults with ID in relation to remote support provided by public support agencies	Qualitative – qualitative. Sample − 22 parents. Data collection – interviews. Data analysis – qualitative. Timeline – May – July 2020.	Remote support offered took a variety of forms, most common forms were video calls and video or audio recordings. Parents had difficulties organizing their time combining their professional, domestic, and therapeutic duties. Most frequently mentioned problems were the lack of contact with friends and how this along with a lack of routine created behavioural problems.	Opportunities for activities was significantly reduced due to material problems such as limited access to a computer or printer.	Little data on qualitative approach and analysis approach.
Yuan et al. (2021) [99] Physical activity and sedentary behaviour among children and adolescents with intellectual disabilities during the COVID‐19 lockdown in China. China.	To explore physical activity and sedentary behaviour among school-aged children and adolescences with ID.	Quantitative – survey research. Sample − 837 children with ID. Data collection - online survey. Data analysis – descriptive and inferential statistics. Timeline – April 2020.	Chinese children and adolescents with ID participated in approximately 10 min of moderate-to-vigorous physical activity and engaged in approximately 530 min of sedentary behaviour every day. Only 17.4% achieved the recommendation 60 min of daily exercise. 76.1% reported adherence to more than 2 h of sedentary behaviour per day.	Decreased physical activity and excessive sedentary behaviour were more prominent in older adolescents compared with younger children with ID.	Convenience sampling used.

ADHD: attention deficit hyperactivity disorder; AHRC: Association for the Help of Retarded Children; ASD: autism spectrum disorder; CAMHS: Child and Adolescent Mental Health Services; CFS: Clinical Frailty Scale; COPD: chronic obstructive pulmonary disease; DS: Down Syndrome; GAD7: generalised anxiety disorder-7; GDPR: General Data Protection Regulation; HA-ID: healthy aging and ID; ID: intellectual disability; LeDeR: Learning Disabilities Mortality Review; PBS: positive behavioural support; PHQ8: Patient Health Questionnaire-8; PPE: personal protective equipment; PTOS-ID II: Psychological Therapy Outcome Scale Second Edition; SMRs: Standardised Mortality Ratios.

### Arranging, summarizing, and communicating the outcomes

The final step involves summarizing and communicating the findings. The findings of this review are presented under the three key questions identified in the first step of the review process. This scoping review generated 84 papers (Table 4) of these 57 were quantitative, 21 qualitative and 6 mixed methods. The papers spanned many countries with 30 from the United Kingdom, 12 from the United States of America, 8 from the Netherlands, 6 from Canada, 5 from Spain, 4 from Poland, 2 each from Saudi Arabia/France/South Korea/China and 1 each from Australia/Austria/Germany/Ireland/Italy/North America, Asia, Europe/Pakistan/South Africa/Turkey/United States of America and Canada/United States of America and Chile.

**Table 4.** Study characteristics.

Research design	Quantitative	[5,17–23,27,30,31,34,35,38–44,46,47,52–55,57–63,65,66,69–72,74,76,77,80–83,85–92,95,96,99]
	Qualitative	[25,28,32,33,41,45,48–51,56,64,67,68,73,75,78,84,94,97,98]
	Mixed methods	[24,26,36,37,79,93]
Country	United Kingdom	[20,22,23,25,27–29,36,37,40,43,44,46,55,64,69,70,75–82,84,92,96,97]
	United States of America	[30,34,38,47,52–54,57,65,87,91,93]
	Netherlands	[32,33,41,45,73,90,93]
	Canada	[51,58–61,83]
	Spain	[18,41,71,72,89]
	Poland	[31,39,74,98]
	Saudi Arabia	[17,21]
	France	[66,86]
	South Korea	[49,50]
	China	[56,99]
	Australia	[19]
	Austria	[88]
	Germany	[95]
	Ireland	[63]
	Italy	[62]
	North America, Asia, Europe	[35]
	Pakistan	[67]
	South Africa	[68]
	Turkey	[48]
	United States of America and Canada	[26]
	United States of America and Chile	[85]

## Results

### What effects are reported by people with intellectual disability and their carers?

COVID-19 effected people with intellectual disability greatly [21], challenges include much higher rates of mortality [27,30,40,53,96], more severe course of the disease [89] and more severe health outcomes [58,91,96]. The most frequent underlying physical health conditions diagnosis were asthma, diabetes, chronic obstructive pulmonary disease (COPD), dementia, cerebral palsy, epilepsy and mental illness and/or addiction [58]. Risk factors for COVID-19 diagnosis and mortality include intellectual disability, multiple comorbidities age, pre-existing conditions, large congregated residential settings and 24 hr skilled nursing staff [27,52–54,91,94,96]. Risks were similar by ethnicity [30]. However, higher rates of medical complications and mortality were associated with people with Down Syndrome [58], especially from age 40 [48]; higher rates of hospital admissions and deaths were also associated with those who have severe to profound intellectual disability and cerebral palsy [40,96]. People with intellectual disability had a higher prevalence of avoidable hospitalizations and multiple comorbidities [91]. The most common reported sign of COVID-19 was fever and frequent respiratory symptoms were dyspnea, hypoxemia and bronchial congestion [86].

Negative effects included changes to routines; activities and social contacts; stress levels and reduced mental well-being; employment, including job losses; restrictions on social contacts and on activities [18,35,36,69,77,85,99]. People with intellectual disability experienced the loss of, reduction in, or significant changes to routines and services received resulting in difficulties accessing services, medical treatment and medication [36,37,85]. People with intellectual disability reported experiencing mental health issues including anxiety and depression; skin picking, obsessive compulsive behaviour, psychotic experiences and suicidal thoughts increased [39,68,95].

However, people with intellectual disability also experienced benefits including enhanced resilience [51]; digital inclusion [36,42,63]; more time spent with people they value; improved health/well-being/fitness; and a slower pace of life [36]. The new relaxed environment resulted in a reduction in challenging behaviours and improvements in sleep, obsessive and compulsive routines, seizures, mood and speech [84]. Social distance and the removal of social pressure created unique opportunities for people with intellectual disability and their families to deepen meaningful relationships and spend quality time with and provide support to their friends, family and caregivers [45,68,71,77]. During lockdown, many people with intellectual disability reported feeling happy, safe and calm and valued support from services [77] and people with Prader Willis Syndrome experienced weight loss [66].

From a carer’s perspective parents focused on protecting their family, by attempting to stay healthy, avoid contacting COVID-19 and adapting to the significant challenges of a dynamic situation as best they could [32,33,49,94]. Parents reported experiencing increased mental health issues including anxiety, stress, distress, loneliness, depression and feeling less emotionally secure [17,48,50,72,75,84]. Parents struggled with social issues such as, financial challenges, food security, the loss of daily routines and healthy behaviours, lack of relevant guidance on COVID-19 regulations and service delivery [17,45,48,50,71,72,76,93,97]. In addition, family demands changed with increased child caring burdens, reduced family and friends support, balancing family and working life, social isolation and increases in behavioural problems [17,45,48,50,71,72,76,93,97], all of which effected family members mental health [84]. Concerns were also expressed by families with regards to lockdowns, reduced or no service provision and poor government funding [67,72] and the negative effect these were having on their child’s health and well-being, their own employment or what would happen if they were unable to care for their child [45,50,67,72]. Parents worried that their adult children may not fully grasp the seriousness of COVID-19, were more vulnerable to the COVID-19 virus, could not identify self-infection and could have fatal outcomes of getting infected with COVID-19 [50]. To manage the varied challenges some parents created house plans [67], while others struggled to maintain appropriate supports for their child and increased their substance misuse and gambling to cope [76]. Siblings expressed anxiety about the health and well‐being of their brother/sister and provided key support within families [83].

The pandemic also created opportunities for families where parents reported experiencing adjustments and hopes [50]. Parents valued the extra time available to bond with their family by sharing meals and playing board games [67,93]. Some parents built up their mental resilience overtime by implementing effective coping strategies such as exercise routines or hobbies [83]. The change in routines and relaxed environment also resulted in less stressful mornings for family members, sleep ins and more rest [93,97]. Resulting in increased sibling well-being and improved behaviour, which reduced stress [84].

At a service level COVID-19 continued to have a profoundly negative effect on service delivery as many closed, reduced provision or transferred partly or wholly to on-line. Neglect by services was also reported [28] with staff experiencing poorer mental health, including stress, anxiety, depression and distress [55,59], with some requiring medication and therapy [59]. Those over 45 were more likely to describe considerable clinical distress compared to younger workers [59]. Mental health issues were due to fears of being infected by clients [57], occupational stress because of the shortage of PPE, difficulties implementing infection control measures; redeployment, lack of access to testing, learning new roles and the use of new technologies without adequate training or support, inadequate tools or equipment to work remotely, demands at home, and changes due to COVID-19 [55,56,92]. All of which effects the quality of support provided to people with intellectual disability in various ways but equally, other staff adopted well to the ever-evolving situation [55,59,78].

### What responses have been directed towards people with intellectual disability and their carers?

Some research identified that staff had the proper PPE to do their jobs safely; and were given the tools, training, and information to reduce risk of infection [57]. Research focused on comparison of case-fatality rates [54], COVID-19 vaccine willingness [29,43,47,60], intention to receive vaccine [29,47] and the fact some did not receive a service [85]. In addition, the research highlighted that consultations, medications and multidisciplinary team input increased per week [80] and an overall increase in psychotropic prescribing during lockdown [70].

The responses evident within the literature are clearly weighted towards adapting service delivery and that professional carers employed varied methods to sustain contact with people with intellectual disabilities during the COVID-19 [33,90]. The use of technologies and novel strategies [19] were clear and adapted to virtual service delivery [25]. Virtual/telehealth methods were positively implemented across a wide range of technology platforms [26,62], enabling the use of online assessment methods [20,65,73], therapy/support sessions [73,74,79,81], virtual service delivery/visits [61,77,87] and support/consultations [70,73,98].

However, there was a clear note of caution in using virtual/telehealth as some clients required carer assistance to access the technology [78] or were unable to engage [82] or did not understand the technology [24] and that providing clients with a photo of the support person needs to be considered to enable an alliance to develop with clients [82]. In addition, in person visits were facilitated where needed [61,77]. Thus, while there is clear use of technology it is not a substitute for face-to-face services/support [64].

Staff adapted to extremely challenging circumstances [28] and service delivery/ways of working [25,78,92]. While technology assisted social networking, service provision [75] and enabled more time to be spent with important people [36] it uses in receiving information about COVID-19 [73] and maintaining social relationships for the quality of life of people with intellectual disability are also recognised [64]. However, it was noted that nurses described being excluded from COVID-19 planning, and there was a deficiency of public health guidelines specific to persons with intellectual/development disability [5].

### What recommendations have been made regarding people with intellectual disability and their carers?

Pandemic planning must ensure people with intellectual disability receive comprehensive services and social supports to enhance their social contact, health and well-being [35,36]. Such responses must ensure access to these essential services [37] and be done in a way that prioritizes the quality of life of the person [38]. There is a need for greater supports from services [17], specialized care [95], support for families [94,95,97] and the involvement of parents in their children’s care [21]. In addition, siblings should be involved in efforts to distribute resources, gain feedback and support mental well-being [83]. Care leavers [68] and migrant families [41] were two groups identified as requiring tailored support and consideration for independent living programme training. As with proper communicative support, individuals can cope effectively with considerable restrictions imposed by a pandemic/lockdown [79,88].

From a practice perspective clinicians should be aware of COVID-19 symptoms in people with intellectual disability [86], health services need to support prompt access to COVID-19 testing [96] and policy/guidance needs to prioritize people with intellectual disability for vaccine [29,91,96]. However, targeted action may be needed to address lower vaccination uptake and vaccination strategies need to address beliefs that the vaccine is unnecessary [61]. Concerns were raised in the research as to the possibility of systemic and professional bias and discrimination affecting treatment decisions for people with intellectual disability [23]. Thus, barriers to care need to be overcome to ensure equity of services [23] and to address care professionals’ attitudes towards, and needs recognition of, people with intellectual disability [31]. In addition, the research highlights that the Clinical Frailty Scale is not suitable to evaluate frailty in individuals with intellectual disability, with potential dramatic consequences for triage and decision-making during the COVID-19 [34]. Furthermore, due to the prevalence of symptoms of anxiety and depression in people with intellectual disability there is a need for screening and psychological support [39] and an urgent requirement for community-based services to address psychosocial challenges [50].

The use of telehealth procedures may be appropriate for a wide range of services and assessments which may ease the burden of travel and eliminate the need for onsite assessment [26]. Telerehabilitation could be a valid means for the rehabilitation of specific cognitive skills [62] and virtual platforms are feasible to deliver therapeutic programmes [87]. However, telehealth is not an option for all as some need face-to-face and other require high level of support to engage [97]. Thus, for some adults with intellectual disability support to improve or gain digital confidence and access is required [36] and support staffs own digital skills are often insufficient [73]. Where client require their carer to help them understand what is being discussed or directed there are confidentiality and engagement issues to be addressed [82].

From a research perspective there is a need for research to identify the ways in which people with intellectual disabilities encounter COVID-19 and experience the disease [44] and longitudinal research to understand the medium-long term effects of COVID-19 on people with intellectual disability, their parental caregivers and their siblings [22]. Furthermore, from a policy perspective intellectual disability nurses must be engaged in public health planning and policy development to safeguard that basic care needs of people with intellectual disability are met [5].

## Discussion

The aim of this scoping review was to update and chart the evidence of the effects of the COVID-19 pandemic on people with intellectual disability, their families and carers reported within the research in 2021. This paper, through identifying the evidence, highlights what we can learn to limit the effect of COVID-19 for people with intellectual disability and deliver suitable care for this population in the current and future pandemics. In this review, a range of experiences are reported related to persons with intellectual disability, their family and carers. This review highlights that people with intellectual disability may be under reflected in national policy, strategy, and the responses to COVID-19 [18] and may be viewed as ‘forgotten within society and the systems around them’ [28, p. 591]. While some countries have intellectual disability reflected in its policy and adopt the 2006 UN Convention of Rights [100] there is a long history of marginalization of people with intellectual disability and their carers, which may have strengthened unabated during the current pandemic. This may be driven by poor attitude towards or limited awareness of intellectual disability. The general population’s attitude towards people with intellectual disability is only slightly positive with adults (35–60 yrs.) articulating the most negative views [31]. What is evident is that the more that individuals know about intellectual disability, the more contact they have with intellectual disability and the type of intellectual disability effects the public’s attitude [101]. From a healthcare professionals’ perspective their attitude to intellectual disability is affected by a fear of the unknown; the complexity of patient needs and the lack of education regarding the health needs of people with intellectual disability [102]. However, it is noted that younger healthcare professionals are more likely to feel that those with intellectual disability should be empowered to take control of their lives whereas older healthcare professionals are more likely to believe that individuals with intellectual disability are vulnerable [103] and that others should speak on their behalf. This suggests a generational difference in attitudes, and the need for educational interventions to improve attitudes among older healthcare professionals to address pre-existing bias/attitudes regarding care provision [103]. However, one must recognize recent advances to support and engage health and social care workforce to better support people with an intellectual disability and autistic such as the Oliver McGowan mandatory training on learning Disability and autism in the UK. While such advances are welcome it is worthy to note this has been in response to highlighted poor care [104].

People with intellectual disability have experienced profound disparities in healthcare, which may have added to excess mortality in this group [23]. The most alarming health disparity recognized for people with intellectual disability is the increased risk of early mortality [105,106]. People with intellectual disability experiences of COVID-19 are excessively higher mortality than that of the general population [93], resulting in a profound effect on their health, quality of life and well-being. As a result, they are particularly vulnerable to the physical, social and mental consequences of the pandemic. Within this review mortality, severity and outcome of COVID-19 were factors for people with intellectual disability and those with underlying conditions. People with Down Syndrome, severity of intellectual disability and being over 40 years of age were more susceptible to Covid-19 and had poorer outcomes [40,41,58]. Therefore, there is a clear need to address the social, health and well-being supports necessary to enable the person with intellectual disability maintain good health. As supports play a vital protective role in facilitating psychosocial outcomes for individuals with intellectual disability there is a need for healthcare practitioners, services and policymakers to provide accessible resources and to develop better support systems to address these gaps [35]. This is essential as this review highlights there was a deficiency of public health guidelines specific to persons with intellectual/development disability and nurses resulting in this group being excluded from much COVID-19 planning [5]. However, thoughtful consideration is warranted regarding the individual supports and resources needed to alleviate the challenges off the COVID-19 crises such as voluntary groups, neighbourhood supports, safe visiting, remote communications [107,108].

This vulnerability the lack of policy, poor attitudes and marginalization can create greater health disparity for people with intellectual disability. Given that this review did not identify the availability of specific public health prevention and protection measures for people with intellectual disability it may be difficult to identify the lessons learned regarding the spread of future viruses and prevention of future global pandemics. While the importance of having accessible information that explains public health prevention and protection measures is accepted [109,110], there are studies indicating people with intellectual disability understood the rules about COVID-19, wearing face masks, shielding, handwashing and social distancing, [18] there is little research evidence examining the accessibility and effectiveness of information and support provided to further help explain the restrictions caused by COVID-19. Accessible information and support are important for people with intellectual disability in relation to COVID-19 vaccine awareness and uptake. While this review highlights a willingness or intention for people with intellectual disability to receive a vaccine [43,47,61]. The question as to whether people with intellectual disability should be prioritized for vaccination [91,96] and the need for targeted activity to address vaccination strategies to address families, professionals and people with intellectual disability beliefs and understanding [61] remains scant. In addition, in terms of vaccination consideration needs to be given to the likelihood of a person with intellectual disability dislike to needles or refusal, which is not to be unexpected within this cohort due to a fear of needles [111,112]. Furthermore, concerns around informed consent and the fact that consent procedures are often found to be inadequate [113] within this cohort makes practice challenging. This literature review found no research addressing this gap in order to develop and share evidence for practice and families.

Generally, within this review it is clear there is a need to recognize and respond to the symptoms of COVID-19 [91], early access to screening [114,115] and a clear need for psychological support through independent living skill programmes and screening for anxiety and depression [39,55,59]. Psychological support needs to consolidate and enhance existing adaptive skills and reduce the negative effects of COVID-19 on the persons emotional well-being and quality of life. Supports appropriate may be around daily routine activities; personal hygiene (safety measures, use of personal protective equipment), clothing and eating (cleanliness of clothing and linen, cutlery, plates and glasses, distancing during meals). Educational interventions to enable understanding of COVID-19, infection and avoidance of infection and procedures adopted (protection equipment, adaptation to the setting, social distancing). Individualized support using modelling, behavioural techniques, peer support/learning and behavioural self-regulation skills. All support information and instruction should be provided utilizing visual aids and verbal instruction that is continually repeated in a clear and accessible language, appropriate to the functioning levels of person. Thus, addressing the attention, memory and learning functions, communication skills, emotional skills, social skills and independence skills of the person.

Evident within this review was that COVID-19 had a clear impact on disability services and saw the introduction of virtual services [116] and this means of support may be preferred over time [117]. As within this review the relationship, accessibility, assistance/support and confidentiality issues were evident which will affect use in the future. Therefore, proceeding with caution is advised as despite the necessity to adopt this form of support while under COVID-19 restrictions, it remains essential to collaborate with the individual as to the approach best suited to supporting them. As the relationship itself is a crucial essential element of in-person support that may assist more effective communication and better enable the development of meaningful therapeutic relationships [61]. The concerns regarding the use and availability of virtual services are justified as concerns regarding confidentiality [118], infrastructure within disability services (availability of reliable broadband and IT/mobile resources) [116], IT literacy of staff, families and persons with intellectual disability [116] and ensuring access, skill, knowledge and resource are meaningful for all [119]. These are real issues within practice and the lives of all concerned [116,118].

The initial scoping review of the 2020 literature [7] in this area identified a flood of opinion pieces and discussions, with only 16 (6%) of 268 papers initially identified in the databases being peer reviewed research papers. While this may have been understandable at the beginning of a pandemic, it is concerning that over two years later the trend continues to grow unabated as shown in the PRISMA flow diagram (Figure 1). Redirecting greater energies to research and less to professional debates and polemic activities may better serve people with intellectual disability and their carers in this and future pandemics. In this updated review, similar to that of the 2020 review of studies [7], a large quantity of the published studies in 2021were undertaken in the early stages of the pandemic therefore the medium-long term effects of COVID-19 are still poorly understood. The authors also note a trend in the number of studies published since 2020, for example on university and organization websites, which have not gone through the academic rigours of peer reviewed publication within the past two years. Furthermore, a number of studies were excluded because they reported links to the pandemic even though their data was gathered pre the existence of COVID-19.

## Limitations

While this review used precise, transparent methods based on study and reporting guidelines [9,14], no quality appraisal was conducted as the focus of this review were to update and map the evidence. Thus, this paper only offers a descriptive account of available information. Furthermore, there was no patient and public involvement and there is an opportunity for engagement, potentially following published guidance on stakeholder involvement in systematic reviews [120]. In addition, most papers in this review describe (1) data that was collected early in the early stages of the pandemic and additional data is essential to identify the medium-long term effects of COVID-19, (2) small sample were often utilized which were not fully representative of the intellectual disability population (socioeconomic, level of disability etc), (3) self-reporting measures were utilized and mainly administered online which limits responses to individuals with internet access and connectivity, and also those with a level of literacy, (4) numerous data collection tools were utilized and may not validated or psychometric proprieties and inferential analysis not conducted, (5) no pre-COVID-19 measures exist for comparisons analysis to be conducted and (6) most research was carried out on people in developed countries resulting in predominantly western care perspectives being promoted and continuing albeit unintentionally the historical trend of excluding the experiences of the vast majority of people with intellectual disability and their cares from the prevailing international narrative.

## Conclusion

Overall, this review reiterates the serious health risk of COVID-19 (health outcomes, hospitalization, mortality), social implications (loss of routines, activities, social contact) and mental health implications (stress, anxiety, obsessive compulsive behaviours) for people with intellectual disability. Positive aspects were identified for families and persons with intellectual disability (enhanced resilience, digital inclusion, valued time with loved ones, well-being, exercise, relaxed). However, families had continually to balance the worries and strains of the pandemic (anxiety, stress, loneliness, financial, carer burden). From a staff and service perspective COVID-19 brought a sudden pivoting of service provision and while the benefits identified were experienced by some this was a time of stress and worry (anxiety, occupational stress, difficulties in implementing infection control measures, shortage of PPE). Of note within this review is the fact that barriers to the use of telehealth are evident (access to technology, access to internet, understanding of technology, support/facilitation). This raise the issue of support needed and confidentiality that needs to be addressed for this vulnerable population. In addition, access and support for vaccination is warranted in relation to accessible information and support strategies to address fear of needles. However, this review highlights the absence of nurses within the decision making, policy framing and implementation of health measures to support people with intellectual disability. Thus, there is little research about supportive strategies for people with intellectual disability in relation to care strategies, health interventions, health promotion, health education, accessible information and vaccination uptake. This review accentuates the need to capture the rich perspectives and experiences of the people with intellectual disability over the medium-long term to understand the specific needs and successful support strategies to ensure a rights-based person-centred approach is prioritized. Healthcare practitioners also need to broaden their awareness and practice to adopt a more collaborative approach to working that includes people with intellectual disability, their family and cares. Moreover, there is a need for public health advice, guidelines and careful application of quality infection control measures for people with intellectual disability.

## Supplementary Material

Supplemental Material

## Data Availability

No data are available other than that reported in this review and available in original published papers used in this review.
